# Activation of STAT3 is a key event in TLR4 signaling-mediated melanoma progression

**DOI:** 10.1038/s41419-020-2440-1

**Published:** 2020-04-20

**Authors:** Xiu-Qiong Fu, Bin Liu, Ya-Ping Wang, Jun-Kui Li, Pei-Li Zhu, Ting Li, Kai-Wing Tse, Ji-Yao Chou, Cheng-Le Yin, Jing-Xuan Bai, Yu-Xi Liu, Ying-Jie Chen, Zhi-Ling Yu

**Affiliations:** 1 Center for Cancer and Inflammation Research, School of Chinese Medicine, Hong Kong Baptist University, Hong Kong, China; 2 Consun Chinese Medicines Research Centre for Renal Diseases, Hong Kong Baptist University, Hong Kong, China; 3 Research and Development Centre for Natural Health Products, HKBU Shenzhen Research Institute and Continuing Education, Shenzhen, China; 4 JaneClare Transdermal TCM Therapy Laboratory, Hong Kong Baptist University, Hong Kong, China

**Keywords:** Melanoma, Melanoma

## Abstract

Malignant melanoma is aggressive and has a high mortality rate. Toll-like receptor 4 (TLR4) has been linked to melanoma growth, angiogenesis and metastasis. However, signal transduction mediated by TLR4 for driving melanoma progression is not fully understood. Signal transducer and activator of transcription 3 (STAT3) has been identified as a major oncogene in melanoma progression. We found: that TLR4 expression positively correlates with activation/phosphorylation of STAT3 in human melanoma samples; that TLR4 ligands activate STAT3 through MYD88 and TRIF in melanoma cells; and that intratumoral activation of TLR4 increases STAT3 activation in the tumor and promotes tumor growth, angiogenesis, epithelial–mesenchymal transition (EMT) and the formation of an immunosuppressive tumor microenvironment in mice. Further, we found that the effects mediated by activating TLR4 are weakened by suppressing STAT3 function with a dominant negative STAT3 variant in melanoma. Collectively, our work identifies STAT3 activation as a key event in TLR4 signaling-mediated melanoma progression, shedding new light on the pathophysiology of melanoma.

## Introduction

Melanoma originated from neural crest-derived melanocytes is an aggressive cancer, with rapid deterioration and high fatality^1^. The pathogenesis of melanoma is complex and is not fully elucidated to date. Although current targeted therapies and immunotherapies for unresectable melanoma are showing exciting clinical results, this disease is still incurable. Understanding the mechanisms of melanoma progression should significantly advance the development of novel therapies for combatting it.

Toll-like receptor 4 (TLR4) is a signaling molecule responsible for clearing pathogens^2^. It is also linked to the development of multiple cancers including melanoma^3^. TLR4 is expressed in 90% of human primary melanoma lesions and 93% of metastatic lesions^4^. In mice, TLR4 has been demonstrated to be an active contributor to UV-induced melanomagenesis^5^. Lipopolysaccharide (LPS), a TLR4 ligand, can increase the proliferation and migration of TLR4-postive melanoma cells but not of TLR4-negative ones^4^. LPS has also been shown to promote lung metastasis of melanoma in mice^6^. These facts together substantiate that TLR4 signaling promotes melanoma development. However, mechanisms underlying TLR4 signaling-mediated melanomagenesis are not fully understood.

Signal transducer and activator of transcription 3 (STAT3), a transcription factor, is constitutively activated in melanoma^7^. Activation/phosphorylation of STAT3 leads to the transcription of a panel of genes involved in melanoma growth, angiogenesis, metastasis and immune evasion^7^. In tumor tissues from colorectal cancer (CRC) patients, it has been found that expressions of TLR4, MyD88 and STAT3 are positively correlated^8^, although whether a pathway involving the three molecules exists and whether it plays a pathogenic role in CRC are not clear. In melanoma, interplay between TLR4 and STAT3 has never been reported.

In this article, we describe four significant findings of our work. First, we found that TLR4 expression and STAT3 activation are positively correlated in human melanoma tissues. Second, we found that TLR4 ligands activate STAT3 through MYD88 (myeloid differentiation primary response gene 88) and TRIF (TIR-domain-containing adapter-inducing interferon-β) in melanoma cells and promote STAT3-mediated cellular events in melanoma progression. Third, we found that constitutive TLR4 signaling enhances STAT3 activation in melanoma tissues and promotes tumor growth, angiogenesis and epithelial–mesenchymal transition (EMT) in mice. Forth, we found that overexpression of a dominant negative STAT3 variant in melanoma cells reverses LPS-provoked tumor growth, angiogenesis and EMT and reprograms melanoma microenvironment in mice. Overall, this study demonstrates that activation of STAT3 is a critical event in TLR4 signaling-mediated melanoma progression.

## Methods

### Human melanoma tissue microarray

Human melanoma tissue microarray (malignant melanoma with skin tissue array, 208 cores, Cat. No. ME2082c) was purchased from US Biomax (MD, USA). Information about age, sex and tumor stages of donors is available on the website https://www.biomax.us/ME2082c. The ethics for collecting human tissue samples and the informed consent of patients provided by the company are available in the supplementary documents. Immunohistochemistry (IHC) staining assays using anti-human TLR4 antibody (SCBT: sc-293072; CA, USA) and anti-human phospho-STAT3 (Y705) antibody (CST: #9145; MA, USA) were performed following standard protocols provided by Servicebio, Inc. (Wuhan Servicebio Technology Co., Ltd., Hubei, China) and scanned using an automatic digital slide scanner (Pannoramic MIDI II, 3D Histech, Ltd., Budapest, Hungary). The sections were semi-quantified using density quant software in the Quant Center (3D Histech), and protein levels were assessed using the histochemistry score (H-score) system.

### Reagents and cell culture

LPS from *Escherichia coli* 0111:B4 were purchased from Sigma-Aldrich (St. Louis, USA). Synthetic lipid A from *Escherichia coli* (MPLAs) was obtained from Invivogen (San Diego, CA, USA). The melanoma cell lines A375 (ATCC® CRL-1619; VA, USA) and B16 (ATCC® CRL-6322), and the human umbilical vein endothelial cell (HUVEC) line (ATCC® CRL-1730) were purchased from American Type Culture Collection (ATCC). Human melanoma cell line IGR-1 was purchased from CLS Cell Lines Service (Eppelheim, Germany). All cell lines had been tested for mycoplasma. A375, B16 and IGR1 cells were cultured in DMEM (Gibco, Thermo Fisher, MA, USA), supplemented with 10% fetal bovine serum (FBS)/1% penicillin-streptomycin (Gibco). The HUVEC cells were cultured in Endothelial Cell Growth Medium (Cell Applications, CA, USA).

To establish stable B16^STAT3β^ and B16^NC^ cell lines, B16 cells were transfected with the pCDNA3.1-3xflag-C-STAT3β construct (FitGene Co., Ltd. Guangzhou, China) and the pCDNA3.1-3xflag-C empty vector (FitGene), respectively, and treated with geneticin to select stably transfected clones^9^. STAT3β lacks a 50 bp domain located near the C terminus compared to the wild-type STAT3. The expression of STAT3 targets Mcl-1, Bcl-xL, VEGF and MMP2 was examined by immunoblotting for confirming the inactivation of STAT3 in the stable B16^STAT3β^ line.

To establish stable A375^CA-TLR4^ and A375^NC^ cell lines, A375 cells were transduced with the lentivirus-pLenti-GFP-Puro-CMV-CA-TLR4 (lentivirus-CA-TLR4, fused with Myc and Flag tags) and the lentivirus-pLenti-GFP-Puro-CMV vector (lentivirus-NC), respectively, and incubated with puromycin for selection of stably transduced clones. The lentivirus-CA-TLR4 and lentivirus-NC were bought from ViGene Biosciences Inc (Shandong, China). The CA-TLR4 fragment was amplified from wide-type TLR4 (NM 138554) lacking DNA sequences encoding the first 20 amino acids (the signal peptide) at N-terminal from a pENTER-TLR4 construct (ViGene Biosciences, CA, USA). TLR4 mRNA level (Fig. S1A) and Myc protein level (Fig. S1B) by RT-qPCR and immunoblotting, respectively, was used to identify the stable lines. The NF-κB and AP-1 transcriptional activities were detected using dual reporter luciferase assays to confirm the activation of TLR4 signaling in the stable A375^CA-TLR4^ line (Fig. S1C).

### Immunoblotting

Cells or tumor tissues were lysed in RIPA lysis buffer containing 50 mM Tris-HCl, 1% NP-40, 0.35% sodium-deoxycholate, 150 mM NaCl, 1 mM EDTA (pH7.4), 1 mM phenylmethylsulfonyl fluoride, 1 mM NaF, 1 mM Na_3_VO_4_ and 10 µg/mL each of aprotinin, leupeptin and pepstatin A. Protein concentrations were measured using the Quick Start™ Bradford Protein Assay (Bio-Rad, CA. USA). Primary antibodies used in this study were STAT3 (CST: #9139), p-STAT3 (Y705; CST: #9145), TLR4 (SCBT: sc-293072), GAPDH (Santa Cruz: sc-32233), Mcl-1 (CST: # 94296), Bcl-xL (CST: # 2764), VEGF (Santa Cruz: sc-507) and MMP2 (CST: # 87809). Immunoblotting was performed following the protocol routinely used in our lab^10^.

### Immunofluorescence staining

A375 cells were treated with LPS (1 µg/mL) for 24 h and then fixed in 4% paraformaldehyde (PFA). The fixed cells were permeabilized using 100% methanol and stained with an anti-STAT3 antibody (CST: #12640; MA. USA) overnight at 4 °C. Cells were then incubated with corresponding secondary antibodies at room temperature for 1 h. Finally, the slices were mounted with DAPI-containing mounting medium (Abcam) and imaged using a fluorescence microscope (Leica DMI3000 B).

### Real-time quantitative polymerase chain reaction analysis

Cells were stimulated with 1 µg/mL of LPS or MPLAs for 48 h. Total RNA of the cells were extracted using Trizol reagent (Thermo Fisher) and reverse transcribed with a reverse transcription kit (Takara, Shiga, Japan) according to manufacturer’s protocol. Real-time quantitative polymerase chain reaction (RT-qPCR) was performed using SYBR green reaction mixture (Bio Rad, PA, USA) in the ViiA 7 real-time PCR system (Applied Biosystems). Primer sequences are shown in Table S1. The gene expression data was normalized to the endogenous control GAPDH. The relative expression levels of genes were calculated according to the formula 2^−ΔΔCt^, where ΔCt is the difference in threshold cycle values between a target and GAPDH, and ΔΔCt = ΔCt sample − ΔCt control^10^.

### Crystal violet staining

To investigate proliferative effects of TLR4 ligands, cells were seeded in the 60 mm dish with 10^4^ cells per well and were incubated with LPS (2 µg/mL) or MPLAs (2 µg/mL) for 7 days (medium was refreshed on day 4). To investigate anti-proliferative effects of parthenolide and TAK-242, cells were seeded in the 60 mm dish with 2 × 10^4^ cells per well and were incubated with parthenolide (1.25, 2.5 µg/mL) or TAK-242 (2.5, 5 µg/mL) for 7 days (medium was refreshed on day 4). At the end of incubation, cells were fixed using 4% paraformaldehyde and stained with 0.1% crystal violet^11^. The culture wells were then washed and photographed. Colony area was measured using ImageJ (1.74 v) software.

### Cell invasion assay

BioCoat™ Matrigel® Invasion Chambers were obtained from Coring (Cat No: #354480; NY, USA). LPS (1 µg/mL)- or MPLAs (1 µg/mL)-treated cells in 350 μL of serum-free DMEM were seeded into the inserts. The lower chambers were filled with 750 μL DMEM supplemented with 10% FBS. After 24 h of incubation, the cells that did not invade through the matrigel were scraped from the insides of the inserts. Cells on the underside of the matrigel were fixed with 4% paraformaldehyde and stained with crystal violet. Cells in three microscope areas were photographed and counted^10,11^.

### Endothelial cell tube formation assay

Supernatant collected from A375 cells after 24 h of LPS (1 µg/mL) or MPLAs (1 µg/mL) stimulation was used as the conditioned media. HUVEC cells were cultured in the conditioned media and plated in 96-well plates coated with 50 μL matrigel/well (BD Bioscience, CA, USA) at the concentration 2 × 10^4^ cells/well for 3 h. Tubes in three microscope areas were photographed by microscopy and counted.

### Human cytokine array

Human Cytokine Array Kit was purchased from R&D Systems (Minneapolis, USA). Conditioned media from A375 cells after 24 h of MPLAs (1 µg/mL) stimulation were subjected to cytokine array assays following the manufacturer’s instructions. The pixel density in each spot of the array was quantified using ImageJ software.

### Enzyme-linked immunosorbent assay (ELISA) assay

Human sICAM-1 (Soluble) Enzyme-linked immunosorbent assay (ELISA) Kit was purchased from ExCell Bio (Shanghai, China). Conditioned media from A375 cells after 24 h of LPS (1 µg/mL) or MPLAs (1 µg/mL) stimulation were subjected to ELISA assays following the manufacturer’s instructions^12^.

### Immunohistochemistry staining

Tumors tissues were fixed in 4% PFA, dehydrated and embedded in paraffin. The paraffin-embedded tissue blocks were cut into 5 μm-thick sections and dewaxed, rehydrated, blocked and then incubated with individual primary antibody at room temperature for 60 min. Primary antibodies used in this study were Ki-67 (Abcam: ab16667), CD31 (Abcam: ab9498), E-cadherin (CST: #3195), N-cadherin (CST: #13116) and Vimentin (CST: #5741). Positive signals were developed using diaminobenzidine (DAB) substrate (Dako Carpinteria, CA, USA) under the manufacturer recommended conditions and photographed^12^. Experiment and result assessment were conducted blind.

### siRNA transfection

The TLR4 siRNAs (238, 604, 905, 1379, and 1555) were bought from GenePharma (Shanghai, China). The MYD88 siRNA (s9138), TRIF siRNA (s531859) and negative control siRNA (4390843) were bought from ThermoFisher. The sequences of these siRNAs are listed in Table S2. A375 cells were transfected with siRNAs using Lipofectamine RNAiMAX Reagent (ThermoFisher). The gene knockdown efficiency was examined using RT-qPCR (Fig. S2).

### Luciferase assay

Dual-Luciferase® Reporter Assay Kit was bought from Promega Corporation (Wisconsin, USA). Cells were seeded in 24-well plates at a density of 5 × 10^4^ cells per well and co-transfected with the STAT3 reporter plasmid 4×M67 pTATA TK-luc (0.2 μg/well, Addgene, USA) and the pRL-CMV vector (0.1 μg/well, promega, USA). After 24 h of transfection, cells were lysed and subjected to dual reporter luciferase assays according to the manufacturer’s instructions. The luminescence of firefly luciferase and the background signal of renilla luciferase were detected using the En Vision Mutilabel Reader (Perkin Elmer, USA)^11^. The transcriptional activity of STAT3 was presented as the luminescence ratio of firefly luc/renilla luc.

### Animal experiments

Male C57/BL6 mice (6-weeks-old) and male *nu/nu* BALB/c mice (6-weeks-old) were bought from the Chinese University of Hong Kong, and maintained in the animal handling room of the Hong Kong Baptist University. All care and handling of animals were performed with the approval of the Research Ethics Committee of Hong Kong Baptist University (Approval number: HASC/15-16/0129). Sample size of each group in our animal studies was calculated based on small-scale studies using the G* power software (Kiel University, version 3.1.9.2). In each calculation, we selected the significant level at 5% (*α* = 0.05) in a two-tailed test and the power at 90% (1 − *β* = 0.9).

To investigate whether constitutive TLR4 signaling promotes melanoma progression, *nu/nu* BALB/c mice were randomly assigned to two groups (*n* = 7; calculated by power analysis based our pilot studies) according to their body weight, and A375^NC^ (1 × 10^6^ cells/mouse) and A375^CA-TLR4^ (1 × 10^6^ cells/mouse) cells were separately subcutaneously (s.c.) injected into the flank of individual mouse. To investigate the role of STAT3 in TLR4 signaling-mediated melanoma progression, C57/BL6 mice were randomly assigned to 4 groups (*n* = 7; calculated by power analysis based our pilot studies) according to their body weight, and B16^NC^ (1 × 10^6^ cells/mouse) and B16^STAT3β^ (1 × 10^6^ cells/mouse) cells suspended in PBS or LPS were separately s.c. injected into the flank of individual mouse. In the two models, mice were euthanized using CO_2_ at day 21. Tumors were collected, weighed and sampled for IHC staining, immunoblotting and flow cytometry immunophenotyping. Spleens from the mice bearing B16^NC^ and B16^STAT3β^ tumors were also collected for immunophenotyping analyses.

To investigate the effects of parthenolide on melanoma tumor growth, B16 (1 × 10^6^) cells were s.c. injected into the flank of individual C57/BL6 mice. At day 7, mice were randomly assigned to four groups (*n* = 5; calculated by power analysis based our pilot studies) according to their tumor sizes and intraperitoneally injected with 0.5, 1, 2 mg/kg/days of parthenolide in 0.2 mL PBS or with the vehicle (0.2 mL of PBS) for 7 consecutive days. At the end of the experiments, mice were euthanized using CO_2_. Tumors were collected, weighed and immunoblotted. A tumor with a volume of <50 mm^3^ at 7 days after cell injection was excluded from the analysis.

### Flow cytometric immunophenotyping

Spleens and tumors from tumor-bearing mice were used to prepare single cell suspensions. Cells incubated with fluorochrome-conjugated antibodies were detected using flow cytometry (12). The anti-CD3 (553063), anti-CD4 (553651), anti-CD8 (551162), anti-NK 1.1 (553164), anti-CD25 (553866), anti-CD86 (553691), anti-CD11c (553802), anti-Gr-1 (561105), anti-CD11b (557397), anti-Foxp3 (563101) and anti-F4/80 (564227) antibodies were bought from BD Biosciences (US). Experiment and data analysis were conducted blind.

### Statistical analysis

Data are presented as mean ± SD unless otherwise indicated in the figure legends. Statistical analyses were carried out using GraphPad Prism version 5.0 (GraphPad Software, San Diego, CA, USA). Pearson correlation and Spearman coefficient were calculated to assess correlation of TLR4 and phosphorylated STAT3 in the human melanoma tissue microarray. Student’s *t*-test was used to compare the difference between two groups. Homogeneity of variance was assessed using Brown-Forsythe test. Comparisons among multiple groups were performed using One-way ANOVA followed by Tukey’s test. *P* < 0.05 was regarded as statistically significant.

## Results

### TLR4 expression and STAT3 phosphorylation are positively correlated in human melanoma samples

**Fig. 1:**
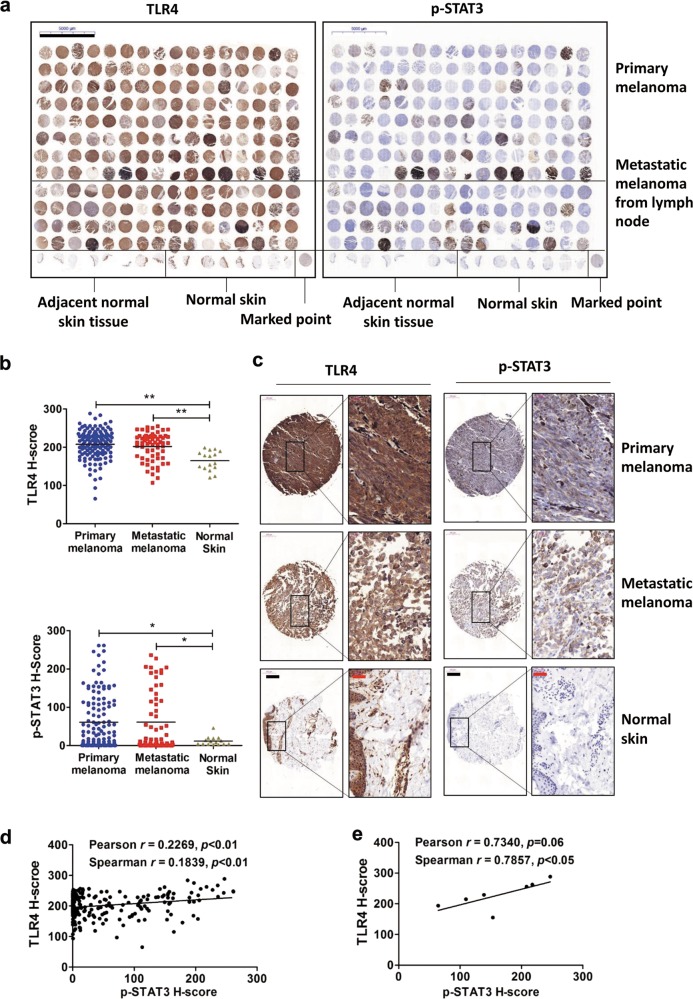
TLR4 expression and STAT3 phosphorylation positively correlate in human melanoma samples. **a** Immunohistochemical (IHC) staining of the expression of TLR4 and phosphorylated STAT3 (p-STAT3; Tyr705) in a human melanoma tissue microarray. Scale bar: 5 mm. **b** Quantification of TLR4 and p-STAT3 staining intensity in the melanoma tissue microarray. *p < 0.05, **p < 0.01. **c** IHC staining of TLR4 and p-STAT3 in representative melanoma specimens and normal skin tissues. Scale bar: black, 200 μm; red, 50 μm. **d** Scatter plots of TLR4 and p-STAT3 immunostaining intensity in human melanoma tissues (*n* = 208). **e** Scatter plots of TLR4 and p-STAT3 immunostaining intensity in early-stage melanoma tissues (*n* = 7).

To determine the correlation of TLR4 expression and STAT3 activation/phosphorylation, we took advantage of the human melanoma tissue microarray (a total of 208 samples) and performed immunohistochemical staining (Fig. 1a). Protein expression levels were semi-quantitatively assessed using the H-score system (Tables S3 and S4). As shown in Fig. 1b, c, protein levels of TLR4 and phosphorylated STAT3 (Y705) were significantly elevated in melanoma tissues compared to that in normal tissues, a finding consistent with previous reports^4,7^. Further analyses showed that TLR4 expression and STAT3 phosphorylation were positively correlated in the 208 samples (Fig. 1d), especially in early-stage (T_1_N_0_M_0_) melanomas (samples C4, D9, F2, F9, G9, H8, H9; Fig. 1e). The positive correlation of TLR4 expression and STAT3 phosphorylation was also found in both male and female patient samples (Fig. S3). In different age groups, TLR4 expression and STAT3 phosphorylation were positively correlated in samples from middle-aged (40–60 years old) and elderly (>60 years old) patients but not in samples from young (<40-years-old) patients (Fig. S3). Reasons for the different observations among patients of <40 years old and ≥40-years-old need to be explored. Collectively, the positive correlation together with the high levels of TLR4 expression and STAT3 phosphorylation in human melanoma tissues suggests a link between TLR4 and STAT3 in melanoma pathogenesis.

### TLR4 ligands activate STAT3 through MYD88 and TRIF in melanoma cells

**Fig. 2:**
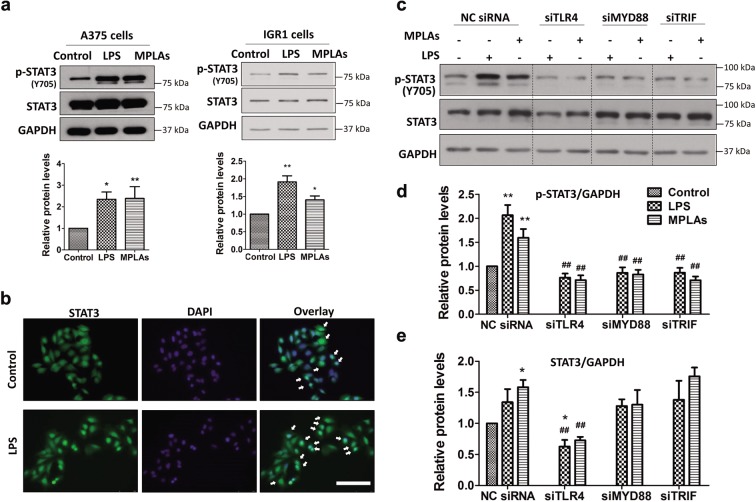
TLR4 ligands activate STAT3 through MYD88 and TRIF in melanoma cells. **a** Immunoblot analyses of total STAT3 and p-STAT3 proteins in LPS- and MPLAs-stimulated melanoma cells. Cells were treated with 1 μg/mL of LPS or MPLAs for 48 h. Representative results (upper panels) and relative protein levels (lower panels) are shown. **b** Immunofluorescence staining of nuclear STAT3 (green) in A375 cells stimulated with LPS (1 μg/mL) for 24 h. DAPI (blue) was used for nuclear staining. Representative images of three independent experiments are shown. Each white arrow denotes the nuclear STAT3. Scale bar: 50 μm. **c–e** Immunoblot analyses of total STAT3 and p-STAT3 proteins in the indicated siRNA-transfected A375 cells. After 48 h of siRNA transfection, cells were treated with 1 μg/mL of LPS or MPLAs for 48 h. Representative results (**c**) and relative protein levels of p-STAT3 (**d**) and total STAT3 (**e**) are shown. Protein levels of p-STAT3 and STAT3 in LPS- and MPLAs-stimulated cells relative to that in cells without TLR4 ligand simulation are regarded as 1. Data are presented as mean ± SD of three independent experiments. *P < 0.05, **P < 0.01 vs. the group without TLR4 ligand stimulation; ^**##**^*P* < 0.01 vs. the individual TLR4 ligand stimulation group of NC siRNA-transfected cells.

To determine whether TLR4 signaling involves STAT3 activation in melanoma cells, we used TLR4 ligands to stimulate the cells and examined STAT3 phosphorylation and nuclear localization. Results showed that both LPS and MPLAs, two TLR4 ligands, increased the phosphorylation of STAT3 in multiple melanoma cell lines (Figs. 2a and S4). Once STAT3 was phosphorylated, it translocates into the nucleus where it functions as a transcription factor^7^. We also found that LPS increased STAT3 nuclear localization in A375 cells (Fig. 2b), further confirming the activation of STAT3 by TLR4 ligand stimulation.

Although MPLAs is claimed to specifically activate TLR4 signaling, LPS activates both TLR4 and TLR2 signaling^13^. TLR4 and TLR2 are widely expressed in melanoma cells^14^. To determine the necessity of TLR4 for LPS- and MPLAs-induced STAT3 activation, we knocked down TLR4 in A375 cells using a specific siRNA (siTLR4; 1555) that has been shown to be able to effectively silence TLR4^15^, and then stimulated the cells with TLR4 ligands. Efficient gene knockdown was confirmed by RT-qPCR (Fig. S2). As shown in Fig. 2c, d, LPS and MPLAs significantly increased STAT3 phosphorylation in A375 cells transfected with the negative control (NC) siRNA. Knockdown of TLR4 completely abolished LPS- and MPLAs-enhanced STAT3 phosphorylation. These data verified that the LPS- and MPLAs-induced STAT3 activation is through TLR4 in melanoma.

Upon ligand recognition, TLR4 signals through MYD88-dependent and/or TRIF-dependent pathways^16^. We found that silencing MYD88 or TRIF, like silencing TLR4, abrogated both LPS- and MPLAs-enhanced STAT3 phosphorylation (Fig. 2c, d). MPLAs is reported to specifically trigger TLR4/TRIF signaling^17^. The observation that knockdown of MYD88 abolishes MPLAs-enhanced STAT3 phosphorylation suggests that TRIF can be regulated by MYD88 in melanoma. Whether MYD88 directly or indirectly regulates TRIF needs to be further investigated. Nevertheless, the findings indicate that both MYD88 and TRIF are involved in TLR4 ligand-mediated STAT3 activation.

In addition, we found that knockdown of TLR4 lowered the protein level but not the mRNA level of STAT3 in melanoma cells (Figs. 2c, e and S5A, B). STAT3 protein degraded faster in siTLR4-transfected cells than in NC siRNA-transfected cells when protein synthesis was blocked by cycloheximide (CHX) in the cells (Fig. S5C, D). MYD88 or TRIF knockdown did not affect total STAT3 protein levels (Fig. 2c, e). These observations suggest that TLR4 signaling uses different mechanisms to activate STAT3 and to sustain STAT3 protein stability.

### TLR4 ligands promote STAT3-mediated cellular events in melanoma progression

**Fig. 3:**
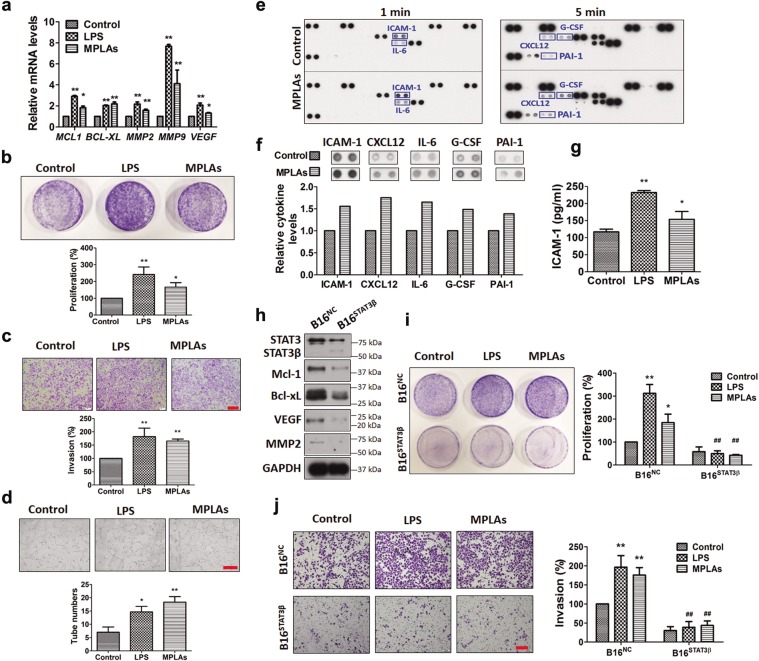
TLR4 ligands promote STAT3-mediated cellular events in melanoma progression. **a** RT-qPCR analyses of *MCL-1*, *BCL-XL*, *MMP2*, *MMP9* and *VEGF* in MPLAs (1 μg/mL, 24 h)- and LPS (1 μg/mL, 24 h)-stimulated A375 cells. **b** TLR4 ligands promote A375 cell proliferation. Cells were treated with 2 μg/mL of LPS or MPLAs for 7 days and stained with crystal violet. **c** TLR4 ligands promote A375 cell invasion. Cells were treated with 1 μg/mL of LPS or MPLAs for 24 h and then subjected to transwell assays. Scale bar: 200 μm. **d** TLR4 ligands promote HUVECs tube formation. HUVECs were incubated with conditioned media from MPLAs (1 μg/mL, 24 h)- or LPS (1 μg/mL, 24 h)-stimulated A375 cells. Scale bar: 500 μm. In **b**–**d**, representative results are shown in the upper panels, and quantitative results of three independent experiments are shown in the lower panels. **e** Human cytokine array analyses of A375 cells with or without MPLAs (1 μg/mL, 24 h) stimulation. The 1- and 5-min exposure results are shown. **f** Quantification of levels of altered cytokines detected in **e**. **g** ELISA analyses of ICAM-1 in conditioned media from MPLAs (1 μg/mL, 24 h)- and LPS (1 μg/mL, 24 h)-stimulated A375 cells. In **a**–**d**, **g**, *P < 0.05, **P < 0.01 vs. control group. **h** Representative immunoblots of STAT3, Mcl-1, Bcl-xL, VEGF and MMP2 in stable B16^NC^ and B16^STAT3β^ cell lines. **i** Photographs of crystal violet-stained B16^NC^ and B16^STAT3β^ cells. Cells were treated as in **b**. **j** Images of invasive cells in the transwell assay. Cells were treated as in **c**. Scale bar: 200 μm. In **i**, **j**, representative results are shown in the left panels; quantification results of three independent experiments are shown in the right panels. *P < 0.05, **P < 0.01 vs. control group of B16^NC^ cells. ^**##**^*P* < 0.01 vs. individual TLR4 ligand stimulation group.

We have observed that TLR4 ligands increase nuclear localization of STAT3 in melanoma cells (Fig. 2b). Here, we examined whether TLR4 ligands affect melanoma progression-related genes that can be transcribed by STAT3 in melanoma cells. RT-qPCR results showed that mRNA levels of *BCL2L1* (*BCL-XL*) and *MCL1* (involved in melanoma cell survival)^7,18^, and *MMP-2*, *MMP-9* and *VEGF* (involved in melanoma metastasis and angiogenesis)^7,19,20^ that can be transcriptionally upregulated by STAT3 were elevated upon LPS or MPLAs stimulation in melanoma cells (Fig. 3a). Next, we examined whether TLR4 ligands trigger the above genes-related malignant behaviors in cell models. Results showed that MPLAs and LPS, in a similar manner, promoted proliferation (Fig. 3b) and invasion (Fig. 3c) of melanoma cells. To test whether TLR4 signaling in melanoma cells promotes angiogenesis, conditioned media from MPLAs- and LPS-stimulated A375 cells were used to separately incubate HUVECs, and formed tubes were counted. Results showed that both conditioned media increased tube formation of HUVECs (Fig. 3d), indicating that TLR4 signaling promotes angiogenesis in vitro.

MPLAs has been clinically tested as an immunotherapeutic agent in treating cancers including melanoma (clinicaltrials.gov Identifier: NCT01584115). We have found that MPLAs activates STAT3 in melanoma cells (Fig. 2). Activation of STAT3 is reported to upregulate immunosuppressive cytokines to promote tumor immune evasion^7^. To examine whether MPLAs provokes the production of immunosuppressive cytokines, we collected the conditioned media from MPLAs-treated A375 cells and conducted a cytokine array assay. Results showed that several immunosuppressive cytokines, including ICAM-1^21^, IL-6^22^, CXCL12^22^, G-CSF^23^, and PAI-1^24^, were increased upon MPLAs stimulation (Fig. 3e, f). G-CSF can be upregulated by TLR4 signaling^25^ and is able to activate STAT3 in melanoma^26^. ICAM-1, IL-6, CXCL12 and PAI-1 are transcriptionally regulated by STAT3^27,28^. ICAM-1 is the most increased in the altered proteins. We quantified it using ELISA assays. Results confirmed that MPLAs significantly increased ICAM-1 secretion; and LPS also increased ICAM-1 secretion (Fig. 3g). Other altered cytokines observed in the cytokine array assay need to be further verified. Notwithstanding, these results show that TLR4 signaling in melanoma cells increases the secretion of immunosuppressive cytokines that can be upregulated by STAT3.

Next, we examined whether activation of STAT3 is required for the cellular events in TLR4 signaling-mediated melanoma progression. For this purpose, we established a pair of stable cell lines, B16^STAT3β^ (a line overexpressing STAT3β) and B16^NC^ (the negative control cell line harbouring the empty vector). STAT3β is a dominant-negative variant of STAT3, containing the dimerization and DNA-binding domains but without the transactivation domain^29^. Immunoblotting showed that protein levels of STAT3 targets Bcl-xL, Mcl-1, VEGF and MMP2 were lower in B16^STAT3β^ cells than in B16^NC^ cells (Fig. 3h), indicating that STAT3 activation is blocked by STAT3β. We found that both LPS and MPLAs significantly increased cell proliferation (Fig. 3i) and invasion (Fig. 3j) in B16^NC^ cells but not in B16^STAT3β^ cells, suggesting a critical role of STAT3 activation in TLR4 signaling-caused melanoma progression.

### Constitutive TLR4 signaling enhances STAT3 activation and promotes melanoma progression in mice

**Fig. 4:**
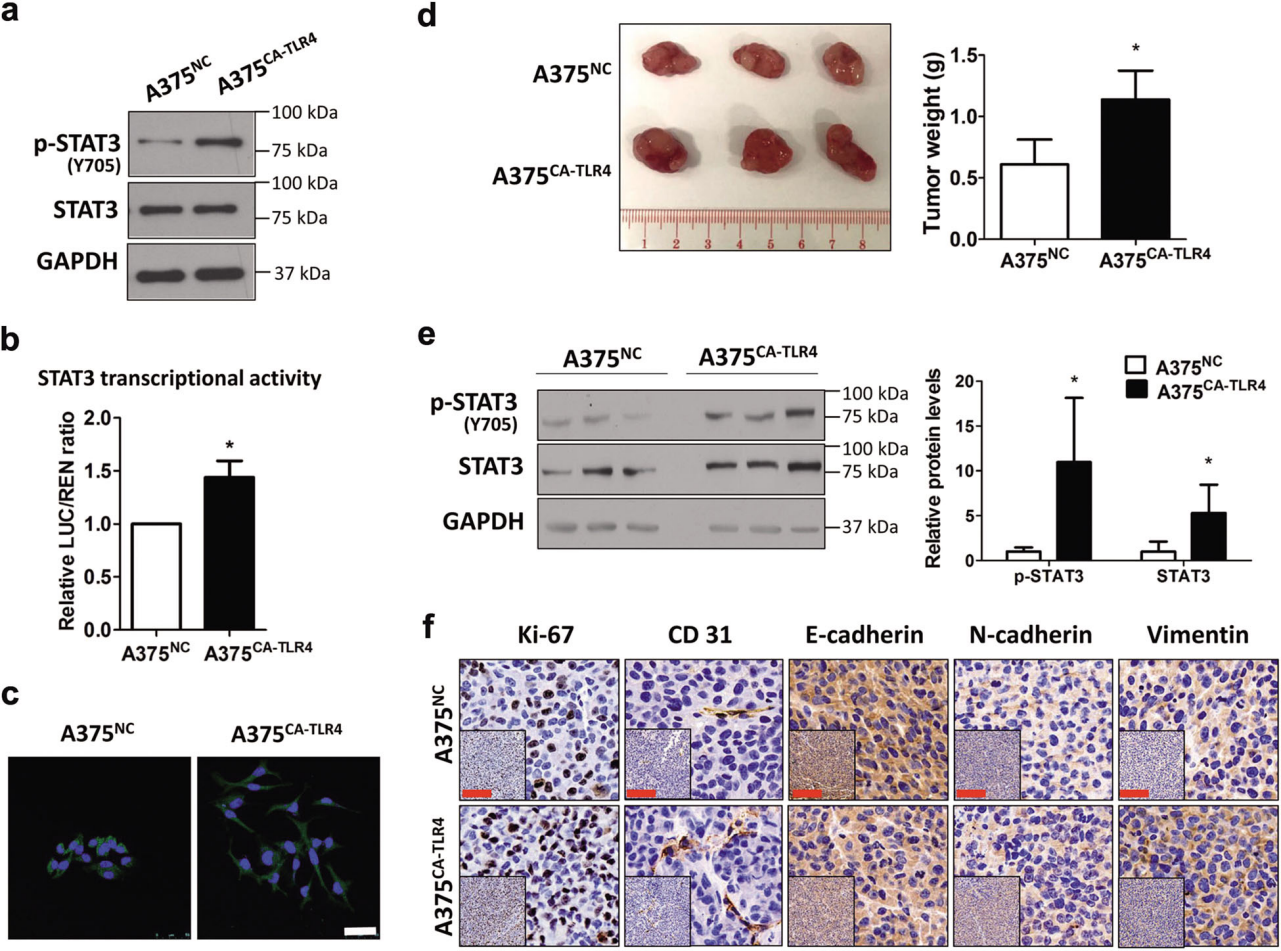
Constitutive TLR4 signaling enhances STAT3 activation and promotes tumor growth, angiogenesis and EMT in mice. **a** Representative immunoblots of total and phosphorylated STAT3 in A375^NC^ and A375^CA-TLR4^ cells. **b** Transcriptional activity of STAT3 in A375^NC^ and A375^CA-TLR4^ cells. **c** Morphological images of A375^NC^ and A375^CA-TLR4^ cells. Cells expressing green fluorescent protein (GFP) displayed green fluorescence. DAPI (blue) was used for nuclear staining. Scale bar: 50 μm. **d** Representative images (left panel) and weights (right panel) of A375^NC^ and A375^CA-TLR4^ tumors (*n* = 7, two independent experiments). **e** Immunoblot analyses of total STAT3 and phosphorylated STAT3 (p-STAT3) in A375^NC^ and A375^CA-TLR4^ tumors. Representative immunoblotting results are shown in the left panel. Protein levels of p-STAT3 and STAT3 in A375^CA-TLR4^ tumors relative to that in A375^NC^ tumors (regarded as 1) are shown in the right panel. GAPDH was used as a loading control. In **a**, **c**–**e**, data are shown as the mean ± SD of three independent experiments. *P < 0.05 vs. A375^NC^ group. **f** Representative IHC staining results of Ki-67, CD31, E-cadherin, N-cadherin and vimentin in A375^NC^ and A375^CA-TLR4^ tumors. Scale bar: 200 μm.

To determine the relation between TLR4 and STAT3 in melanoma in vivo, we established a pair of stable cell lines, A375^CA-TLR4^ (harbouring a constitutively active variant of TLR4) and A375^NC^ (the negative control cell line harbouring the empty vector), then compared STAT3 activity in the two lines, and further compared tumor growth, angiogenesis and EMT in A375^CA-TLR4^- and A375^NC^-bearing mice. Higher transcriptional activity of two TLR4 downstream transcription factors NF-κB and AP-1 (Fig. S1C) in A375^CA-TLR4^ cells than in A375^NC^ cells confirmed constitutive TLR4 signaling in A375^CA-TLR4^ cells. It was found that protein level of phosphorylated STAT3 and transcriptional activity of STAT3 were higher in A375^CA-TLR4^ cells than in A375^NC^ cells (Fig. 4a, b), indicating that constitutive activation of TLR4 enhances STAT3 activation in melanoma cells. EMT is an early event of tumor cell invasion and metastasis^30^. EMT-like morphological features, such as losing cell-cell interaction and a spindle-shaped phenotype, were observed in A375^CA-TLR4^ cells (Fig. 4c), suggesting that TLR4 activation promotes EMT in melanoma. In A375^CA-TLR4^ and A375^NC^ xenografts-bearing mouse models, weights of A375^CA-TLR4^ xenografts were heavier than those of A375^NC^ xenografts (Fig. 4d). Protein levels of total and phosphorylated STAT3 were upregulated in A375^CA-TLR4^ tumors (Fig. 4e). IHC staining of tumor tissues showed that protein levels of Ki-67 (a cell proliferation marker), CD31 (an angiogenic marker), N-cadherin and vimentin (two mesenchymal markers) were increased and E-cadherin (an epithelial marker) was decreased in A375^CA-TLR4^ tumors compared to A375^NC^ tumors (Fig. 4f). Together, these data indicate that activation of TLR4 enhances STAT3 activation in melanoma tissues and promotes tumor growth, angiogenesis and EMT in mice.

### Activation of STAT3 is required for TLR4 signaling-mediated melanoma progression in mice

**Fig. 5:**
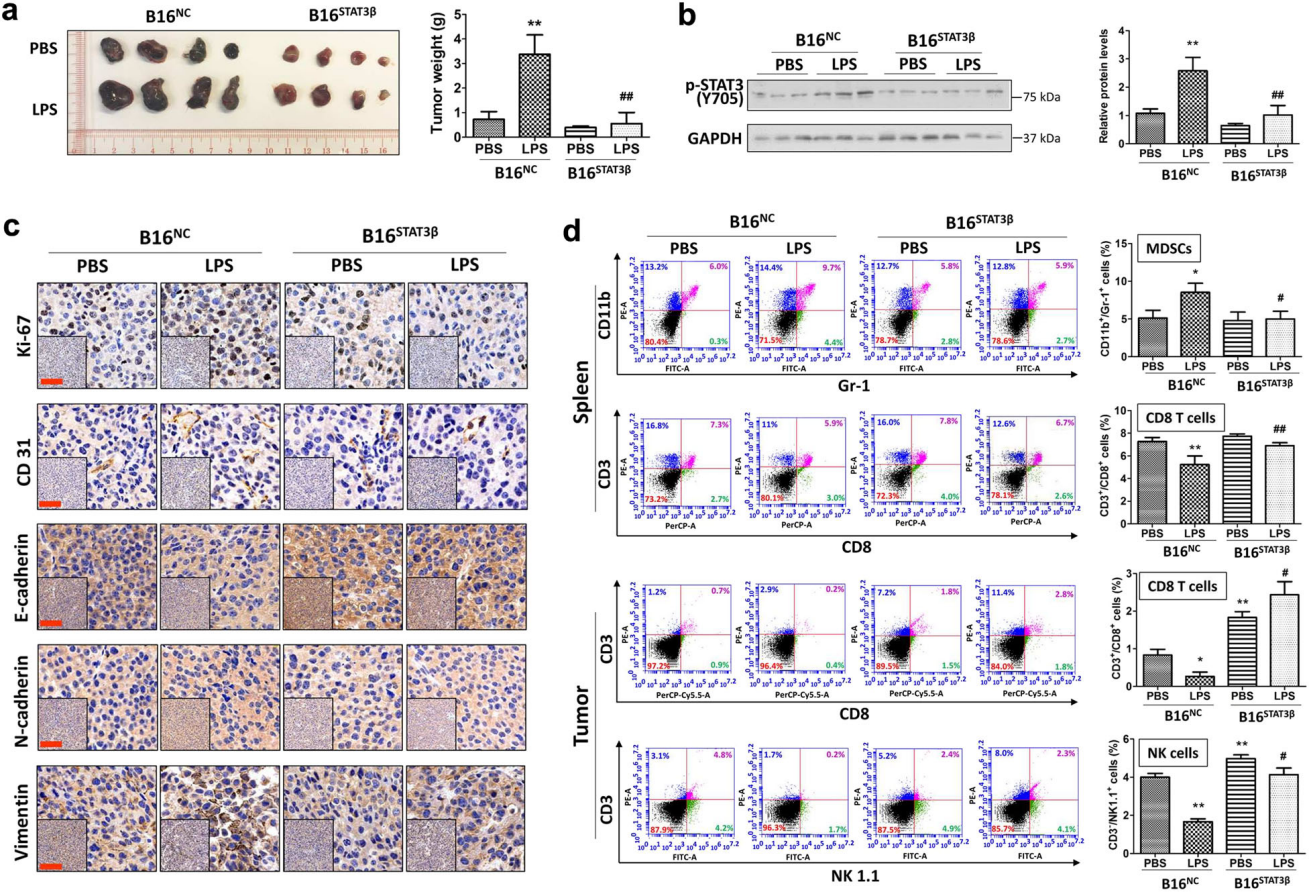
Overexpression of a dominant negative form of STAT3 attenuates TLR4 signaling-mediated melanoma progression in mice. **a** Representative images (left panel) and weights (right panel) of B16^NC^ and B16^STAT3β^ tumors (*n* = 7, two independent experiments). **b** Immunoblot analyses of phosphorylated STAT3 (p-STAT3) in B16^NC^ and B16^STAT3β^ tumors. Representative immunoblotting results are shown in the left panel. Relative protein levels of p-STAT3 in tumors are shown in the right panel. The protein level of p-STAT3 in B16^NC^ tumors without LPS stimulation was regarded as 1. **c** Representative IHC staining results of Ki-67, CD31, E-cadherin, N-cadherin and vimentin in B16^NC^ and B16^STAT3β^ tumors. Scale bar: 200 μm. **d** Flow cytometric analyses of splenic immune cells and tumor-infiltrating immune cells in the melanoma-bearing mice. Representative flow cytometry plots are shown in the left panels. Percentages of splenic MDSCs and CD8 T cells, and tumor-infiltrating CD8 T and NK cells are shown in the right panels. Data are shown as the mean ± SD. *P < 0.05 vs. the B16^NC^ tumor group without LPS stimulation. ^**#**^*P* < 0.05, ^**##**^*P* < 0.01 vs. the LPS-stimulated B16^NC^ tumor group.

Next we examined whether activation of STAT3 is required for TLR4 signaling-mediated melanoma progression in vivo. B16^NC^ and B16^STAT3β^ cells suspended separately in PBS in the presence or absence of LPS were subcutaneously injected into the flank of individual C57BL/6 mice. On day 21 after cell injection, mice were euthanized, and tumors were excised and weighed. Similar to previous reports, the B16^STAT3β^ tumors were smaller and lighter than the B16^NC^ tumors (Fig. 5a). We further found that LPS promoted tumor growth (Fig. 5a) and STAT3 activation (Fig. 5b) in B16^NC^ tumors, but had less effect in B16^STAT3β^ tumors, indicating that STAT3 activation plays a positive role in TLR4 signaling-mediated melanoma growth. IHC staining of tumor tissues showed that expression levels of Ki-67, CD31, N-cadherin and vimentin were increased, while the expression of E-cadherin was decreased in LPS-stimulated B16^NC^ tumors, compared to that in B16^NC^ tumors without LPS stimulation (Fig. 5c). No significant differences were observed in Ki-67, CD31, E-cadherin, N-cadherin and vimentin expression in B16^STAT3β^ tumors with and without LPS stimulation (Fig. 5c). The results of IHC staining imply an important role of STAT3 activation in TLR4 signaling-mediated melanoma growth, angiogenesis and EMT.

Activation of STAT3 has been shown to contribute to tumor-associated immunosuppression^7^. We found that LPS stimulation increased the percentage of splenic myeloid-derived suppressor cells (MDSCs; CD11b^+^Gr-1^+^ cells) in B16^NC^ tumor-bearing mice but not in B16^STAT3β^ tumor-bearing mice (Fig. 5d). MDSCs is responsible for immunosuppression that restricts the activity and tumor infiltration of cytotoxic CD8 T cells^31^. Percentages of splenic and tumor-infiltrating CD8 (CD3^+^CD8^+^) T cells were significantly decreased in B16^NC^ tumor-bearing mice upon LPS stimulation (Fig. 5d). In B16^STAT3β^ tumor-bearing mice, LPS had a less potent decreasing effect on the number of splenic CD8 T cells than in B16^NC^ tumor-bearing mice (Fig. 5d), suggesting a role of STAT3 activation in LPS-induced splenic CD8 T cell reduction. Of note, compared to PBS, LPS increased tumor-infiltrating CD8 T cells in B16^STAT3β^ tumor-bearing mice. This might be because LPS triggers TLR4/TRIF/type I interferon (IFN) signaling and subsequently upregulates IFNs^32^ to enhance CD8 T cell infiltration into tumors^33^. Like CD8 T cells, natural killer (NK) cells directly kill cancer cells^34^. Flow cytometric analyses showed that the number of NK (NK1.1^+^CD3^-^) cells was obviously decreased in LPS-stimulated B16^NC^ tumors, but not in B16^NC^ tumors without LPS stimulation (Fig. 5d). Blocking STAT3 activation by expressing STAT3β in the tumors increased tumor-infiltrating NK cells and attenuated LPS-induced NK cell reduction (Fig. 5d). The changes of immune cell profiles imply that activation of STAT3 contributes to TLR4 signaling-mediated immunosuppression in melanoma microenvironment.

### Pharmacological inhibition of the TLR4/STAT3 pathway restrains melanoma cell proliferation in vitro and in vivo

**Fig. 6:**
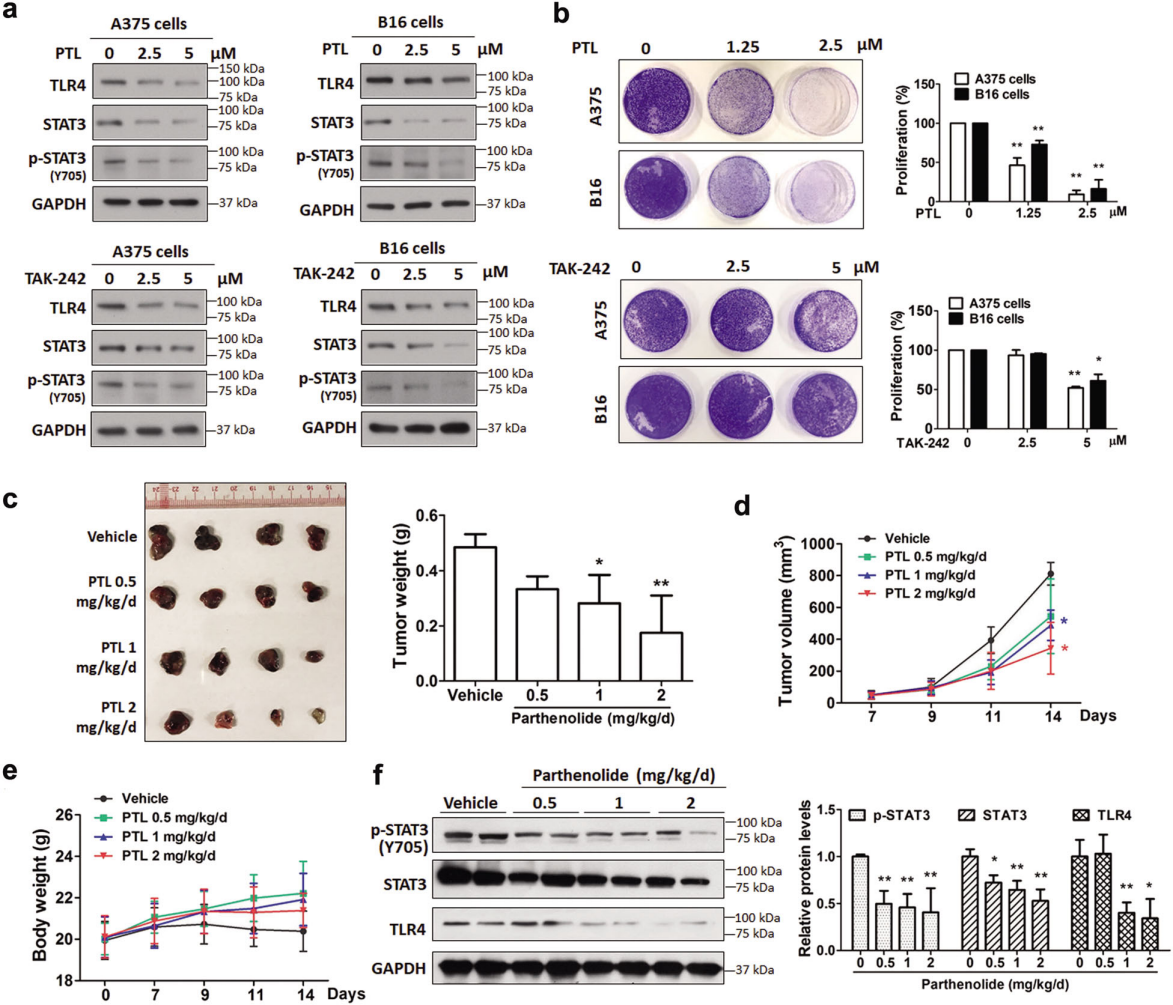
Pharmacological inhibition of the TLR4/STAT3 pathway restrains melanoma cell proliferation in vitro and in vivo. **a** Immunoblot analyses of TLR4, total STAT3 and p-STAT3 proteins in parthenolide (PTL)- and TAK-242-treated melanoma cells. Cells were treated with parthenolide or TAK-242 for 24 h. Representative results of three independent experiments are shown. GAPDH was used as a loading control. **b** Pharmacological inhibition of the TLR4/STAT3 pathway inhibit proliferation of melanoma cells. Cells were treated with parthenolide or TAK-242 for 7 days and stained with crystal violet. Photographs of stained cells are shown in the left panel. Quantitative results are shown in the right panel. The area of cell colonies in the control group was regarded as 100%. *P < 0.05, **P < 0.01, vs. the individual control group. **c** Parthenolide suppresses tumor growth in melanoma-bearing mice. Representative images of B16 tumors are shown in the left panel. Weights of tumors are shown in the right panel. *P < 0.05 vs. vehicle (PBS)-treated control group. **d** B16 tumor volumes. *P < 0.05 vs. vehicle-treated control group at each time point. **e** Body weights of B16 tumor-bearing mice. Data are shown as the mean ± SD, *n* = 5. **f** Parthenolide inhibits the TLR4/STAT3 pathway in tumor tissues from melanoma-bearing mice. Immunoblot analyses of TLR4, STAT3 and phosphorylated STAT3 (p-STAT3) in B16 tumors. Representative immunoblotting results are shown in the left panel. Relative protein levels of p-STAT3, STAT3 and TLR4 are shown in the right panel. Data are presented as mean ± SD of three independent experiments. *P < 0.05 vs. vehicle-treated control group.

We have shown that STAT3 activation is important for TLR4 signaling-mediated melanoma progression. Next, we examined whether inhibiting the TLR4/STAT3 pathway suppresses proliferation of melanoma cells in vitro and in vivo. Parthenolide, a natural sesquiterpene lactone that inhibits TLR4^35^ and STAT3^36^ pathways in multiple cancer cells, and TAK-242, a TLR4 antagonist, were used here. Results showed that both parthenolide and TAK-242 dose-dependently lowered protein levels of TLR4, STAT3 and phosphorylated STAT3 (Fig. 6a) in and inhibited the proliferation (Fig. 6b) of A375 and B16 melanoma cells. To corroborate the in vitro data, we examined the effects of parthenolided on tumor growth in B16 melanoma-bearing mice. As shown in Fig. 6c–e, parthenolide dose-dependently decreased tumor weight and tumor volume, without affecting the body weight of mice. Immunoblotting data showed that parthenolide significantly lowered protein levels of TLR4, STAT3 and phosphorylated STAT3 in B16 tumors (Fig. 6f). These results suggest that inhibiting the TLR4/STAT3 pathway is a viable strategy for treating melanoma.

## Discussion

**Fig. 7:**
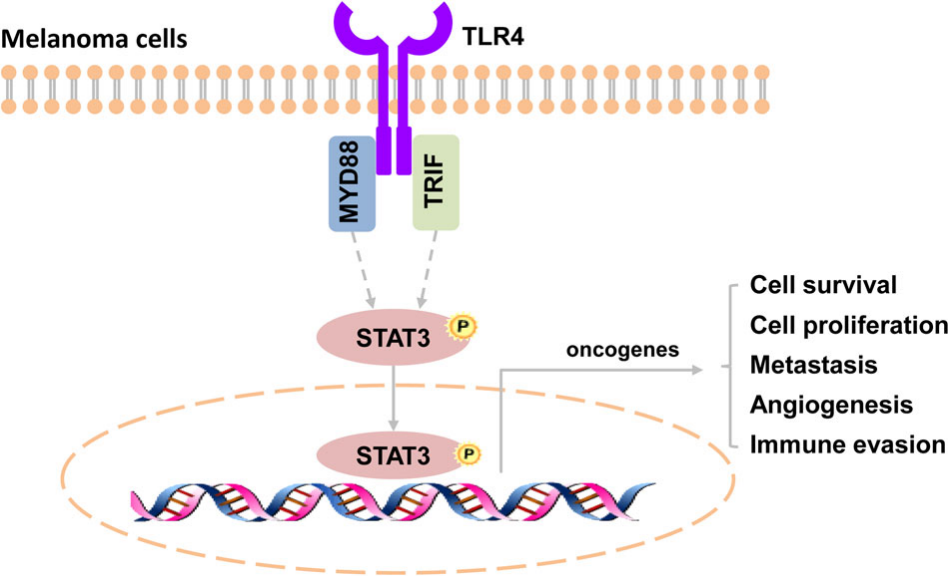
TLR4/STAT3 signaling pathway in melanoma. TLR4 signaling activates STAT3 through MYD88 and TRIF, and subsequently upregulates a series of STAT3 target genes to promote melanoma progression.

In this study, our findings provide evidence that STAT3 is a key player in TLR4 signaling-mediated melanoma progression. TLR4 signaling activates STAT3 through MYD88 and TRIF, and subsequently upregulates a series of STAT3 target genes to promote melanoma progression (Fig. 7).

Studies have shown the contribution of TLR4 signaling in melanomagenesis. Takazawa et al. reported that LPS triggers TLR4/MYD88 signaling to promote migration of melanoma cells^4^. Bald et al. demonstrated that ultraviolet (UV)-radiation induces TLR4/MYD88 axis-driven neutrophilic inflammation to promote melanoma angiogenesis and metastasis^5^. The study done by Yu *et al*. showed that LPS and a TLR4 endogenous ligand HMGB1 trigger TLR4 signaling to activate platelets, and thus promote melanoma metastasis^6^. In this study, we demonstrated that activation of TLR4/MYD88/STAT3 and TLR4/TRIF/STAT3 pathways promotes proliferation of cultured melanoma cells. Moreover, in a mouse model, we demonstrated that STAT3 activation is required for TLR4 signaling-promoted melanoma progression in mice. We are interested in determining whether MYD88 and TRIF are involved in TLR4 signaling-mediated STAT3 activation in vivo in the future.

Receptor signaling can be constitutively activated by mutation(s) of the receptor and/or mutation(s) of molecule(s) in its signaling pathway(s)^37^. It is reported that there are 6.1% of patients with cutaneous melanoma harboring *TLR4* non-synonymous mutations^38^. But the functions of the mutated *TLR4* in melanoma are still unknown. No mutation of *MYD88*, *TRIF* and *STAT3* genes has been reported in melanoma patients. Our paraffin-embedded melanoma samples, purchased from US Biomax, Inc., cannot be used to detect gene polymorphisms. We can collect additional patient melanoma samples to study the roles of gene polymorphisms of *TLR4*, *MYD88*, *TRIF*, and *STAT3* in melanomagenesis in the future.

In the present study, we found that protein levels of TLR4 were significantly elevated in melanoma tissues compared to that in normal tissues. We also found that activation of STAT3 is a key event in TLR4 signaling-mediated melanoma development. PU.1 has been reported to be responsible for TLR4 transcription^39^, and has been identified as a transcriptional target of STAT3^40^. We therefore speculate that there is a TLR4/STAT3/PU.1 positive feedback loop in melanoma, which leads to the high expression of TLR4 and constitutive activation of STAT3. Further studies are required for establishing the TLR4/STAT3/PU.1 loop in melanoma.

In the tissue microarray analyses, we found that TLR4 expression and STAT3 phosphorylation are positively correlated in melanoma tissues. Although more than 90% of cells detected in clinical melanoma specimens are cancer cells^41^, there is a certain percentage of immune cells, such as T cells, DCs and macrophages, in the specimens. The TLR4 ligand LPS has been reported to activate STAT3 in DCs^42^ and macrophages^43^. Whether TLR4 expression is positively correlated with STAT3 activation in tumor-infiltrating immune cells is a question to be addressed.

It is known that, upon ligand binding, TLR4 recruits MYD88 and TRIF through Mal and TRAM, respectively, to activate downstream IKK/NF-kB, MAPK/AP1 and TKB1/IRF3 pathways^44^. In addition to MYD88 and TRIF, other molecules might be involved in the signaling cascades from TLR4 to STAT3 in melanoma. In pilot studies, we have found that inhibitors of IKK (BMS-345541), JNK (SP600125) and TLR4 (TAK-242), but not inhibitors of ERK (U0126) and p38 (SB203580), block LPS-induced STAT3 activation (Fig. S6), suggesting that IKK and JNK are involved in TLR4-signaling-mediated STAT3 activation in melanoma. To delineate the comprehensive molecular events involved in TLR4-mediated STAT3 activation, further studies are needed.

The TLR4/TRIF pathway, responsible for interferon production, is thought to promote innate immune responses^45^. MPLAs, a ligand that triggers TLR4/TRIF signaling^14^, has been used as an immunotherapy in clinical trials for treating cancers including melanoma^46^. In this study, we showed that MPLAs activates STAT3 in melanoma cells, promotes melanoma cell proliferation, invasion and immunosuppressive cytokine secretion, and enhances pro-angiogenic effects of melanoma cells. Our findings suggest that caution should be taken when using MPLAs or other TLR4/TRIF pathway activators to treat melanoma. Combinational use of a TLR4/TRIF pathway activator and a STAT3 inhibiting agent may be a solution; however, further testing is required before clinical use.

STAT3 is a well-established target in experimental melanoma treatment^7^. STAT3 inhibitors that are being used in clinical trials exert anti-melanoma effects by inhibiting STAT3 activation^47^. However, their clinical efficacy is not ideal^47^. Up to now, no STAT3 inhibitor has been approved as a drug. Lowering STAT3 protein level may be able to more effectively block STAT3 signaling. Current strategies are to block STAT3 mRNA translation using decoy oligodeoxynucleotide (ODN) and antisense oligonucleotide (ASO)^48^. Unfortunately, physicochemical properties of ODN and ASO limit their clinical development^48^. In this study, we found that knockdown of TLR4 not only inhibits STAT3 activation, but also induces STAT3 protein degradation, suggesting an alternative approach to blocking STAT3 signaling in melanoma: genetically or pharmacologically inhibiting TLR4.

Parthenolide has anti-inflammatory and anticancer activities. It downregulates TLR4 expression in THP-1 monocytes^49^ and inhibits activation of NF-κB, a TLR4 downstream transcription factor, in melanoma cells^50^. In addition, parthenolide inhibits STAT3 activation and exerts anticancer effects in breast cancer, gastric cancer, prostate cancer and colon cancer cells^36,51^. Whether parthenolide affects both TLR4 and STAT3 in melanoma has not been reported. In this study, we showed that parthenolide, like the TLR4 antagonist TAK-242, lowers protein levels of TLR4, STAT3 and phosphorylated STAT3 in, and inhibits the proliferation of, melanoma cells. Parthenolide also downregulates protein levels of TLR4, STAT3 and phosphorylated STAT3 in tumor tissues and impedes tumor growth in melanoma-bearing mice. These findings indicate that targeting the TLR4/STAT3 pathway is a viable strategy for treating melanoma.

In this study, we established TLR4/MYD88/STAT3 and TLR4/TRIF/STAT3 pathways in melanoma and demonstrated their pathogenic roles. TLR4 expression and constitutive STAT3 activation are also found in other cancers, such as liver^52^, lung^53,54^, and stomach cancers^55^. In an attempt to extend our observations to other cancer types, we analyzed the correlation of TLR4 expression and STAT3 phosphorylation in human liver cancer, lung cancer and stomach cancer tissues using tissue microarray assays. In contrast to a previous report showing that TLR4 expression and STAT3 phosphorylation are positively correlated in primary tumors from liver cancer patients (13 cases)^52^, we found their correlation not significant in liver carcinoma tissues from the microarray (120 cases; BC03119b, US Biomax; Fig. S7A). These different results regarding the correlation between TLR4 expression and STAT3 activation in liver cancer may be because of the different sample sizes of the two analyses. Similarly, no significant correlation between TLR4 expression and STAT3 activation was observed in human lung cancer tissues (120 cases; BC041115d, US Biomax; Fig. S7B) and in stomach cancer tissues (102 cases; ST1021, US Biomax; Fig. S7C) from the microarrays. LPS has been shown to activate STAT3 signaling in cervical cancer^56^ and bladder cancer cells^57^. Whether TLR4 signaling activates STAT3 through the MYD88 and TRIF pathways in the two cancer types, as we observed in melanoma, and whether TLR4/STAT3 signaling plays a pathogenic role in the two cancers warrant further investigations.

In conclusion, activation of STAT3 is a key event in TLR4 signaling-mediated melanoma progression. The TLR4/MYD88/STAT3 and TLR4/TRIF/STAT3 pathways established in this study shed novel insights into the pathophysiology of melanoma, and suggest new, potentially more effective, targets for treating melanoma.

## Supplementary information

Supplementary Figure legends

Supplementary Figure S1

Supplementary Figure S2

Supplementary Figure S3

Supplementary Figure S4

Supplementary Figure S5

Supplementary Figure S6

Supplementary Figure S7

Supplementary Tables
