# Supplementary material for: Activation of STAT3 is a key event in TLR4 signaling-mediated melanoma progression
Source: Cell Death Dis. 2020 Apr 20;11(4):246. doi: 10.1038/s41419-020-2440-1 (PMC7171093; doi:10.1038/s41419-020-2440-1)
Supplement: Supplementary file 1 — Supplementary Figure legends [file 41419_2020_2440_MOESM1_ESM.docx]

**Figure S1.** TLR4 mRNA level, Myc protein level, NF-kB and AP-1 transcriptional activities in stable A375^NC^ and A375^CA-TLR4^ lines. (**A**) RT-qPCR analyses of *TLR4* in the stable lines. *GAPDH* was used as an endogenous control. ** *P* < 0.01 *vs*. the A375^NC^ group. (**B**) Immunoblot analyses of Myc-tag protein in the stable lines. Representative results of three independent experiments are shown. GAPDH was used as a loading control. **(C)** Transcriptional activities of NF-κB and AP-1 in A375^NC^ and A375^CA-TLR4^ cells. Data are shown as the mean ± SD. * *P* < 0.05 vs. A375^NC^ group.

**Figure S2.** RT-qPCR analyses of mRNA levels of TLR4, MYD88 and TRIF in NC siRNA-, siTLR4 (1555)-, siMYD88- and siTRIF-transfected A375 cells. Cells were subjected to RT-qPCR analyses after 48 h of transfection.

**Figure S3.** Correlation analyses of TLR4 expression and STAT3 phosphorylation in human melanoma samples from different sex and age groups. Scatter plots of TLR4 and p-STAT3 immunostaining intensity in the tissues are shown.

**Figure S4.** Immunoblot analyses of total STAT3 and p-STAT3 proteins in TLR4 ligand-stimulated melanoma cells. **(A)** LPS time-dependently increased STAT3 phosphorylation in A375 and B16 cells. Cells were treated with 1 μg/mL of LPS. **(B)** LPS and MPLAs increased the phosphorylation of STAT3 in human melanoma cells. Cells were treated with 1 μg/mL of LPS or MPLAs for 48 h. Representative results of three independent experiments are shown in upper panels. Protein levels of p-STAT3 in TLR4 ligand-stimulated cells relative to that in cells without TLR4 ligand simulation (regarded as 1) are shown in the lower panels. * *P* < 0.05, ** *P* < 0.01 *vs*. the group without TLR4 ligand stimulation.

**Figure S5** Knockdown of TLR4 leads to STAT3 protein degradation in melanoma cells. **(A)** Immunoblot analyses of TLR4, STAT3 and phosphorylated STAT3 proteins in siTLR4-transfected cells. Cells were lysed and immunoblotted after 72 h of siRNA transfection. **(B)** RT-qPCR analyses of mRNA levels of *TLR4* and *STAT3* in NC siRNA- and siTLR4 (1555)-transfected A375 cells. *GAPDH* was used as an endogenous control. Data are presented as mean ± SD of three independent experiments. * *P* < 0.05, ** *P* < 0.01 *vs*. the NC siRNA transfected cells. **(C)** Time course study for STAT3 protein degradation. After 48 h of siRNA transfection, cycloheximide (CHX, 100 μg/mL) was added to A375 cells at the indicated time points and immunoblotted. **(D)** STAT3 protein level percentages at indicated time points with that at 0 h regarded as 100%. Data are presented as mean ± SD of three independent experiments. The dashed line indicates the half-life (t_½_) of the STAT3 protein in A375 cells.

**Figure S6.** Inhibitors of IKK (BMS-345541), JNK (SP600125) and TLR4 (TAK-242), but not inhibitors of ERK (U0126) and p38 (SB203580), block LPS-induced STAT3 activation in A375 cells. Cells were pre-treated with BMS-345541 (2.5 μM), U0126 (1 μM), SB203580 (10 μM), SP600125 (10 μM) and TAK-242 (5 μM) for 1 h, followed by 1 μg/mL of LPS stimulation for 48 h. Representative immunoblotting results of three independent experiments are shown. GAPDH was used as a loading control.

**Figure S7.** Correlation analyses of TLR4 expression and STAT3 phosphorylation in human liver carcinoma, lung cancer and stomach cancer tissues. (**A**) Immunohistochemical (IHC) staining of the expression of TLR4 and phosphorylated STAT3 (p-STAT3; Tyr705) in a human liver carcinoma tissue microarray (120 cases; BC03119b, US Biomax) are shown in left panel. Scatter plots of TLR4 and p-STAT3 immunostaining intensity in the liver carcinoma tissues are shown in right panel. (**B**) IHC staining of the expression of TLR4 and p-STAT3 in a human lung cancer tissue microarray (120 cases; BC041115d, US Biomax) are shown in left panel. Scatter plots of TLR4 and p-STAT3 immunostaining intensity in the lung cancer tissues are shown in right panel. (**C**) IHC staining of the expression of TLR4 and p-STAT3 in a human stomach cancer tissue microarray (102 cases; ST1021, US Biomax) are shown in left panel. Scatter plots of TLR4 and p-STAT3 immunostaining intensity in the stomach cancer tissues are shown in right panel. Scale bar: 4 mm.
