# Supplementary figures and images for: Activation of STAT3 is a key event in TLR4 signaling-mediated melanoma progression
Source: Cell Death Dis. 2020 Apr 20;11(4):246. doi: 10.1038/s41419-020-2440-1 (PMC7171093; doi:10.1038/s41419-020-2440-1)

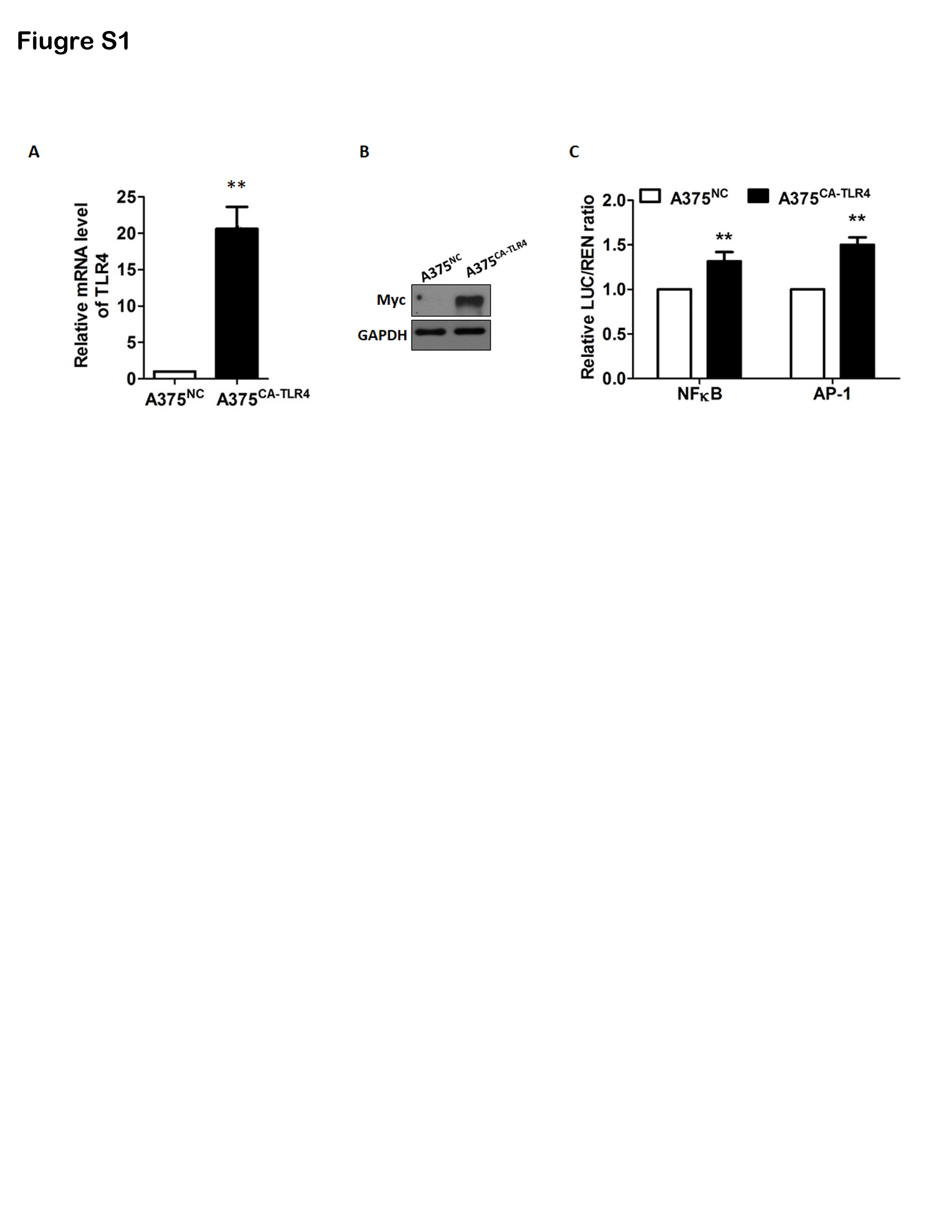

Supplement: Supplementary file 2 — Supplementary Figure S1 [file 41419_2020_2440_MOESM2_ESM.png]

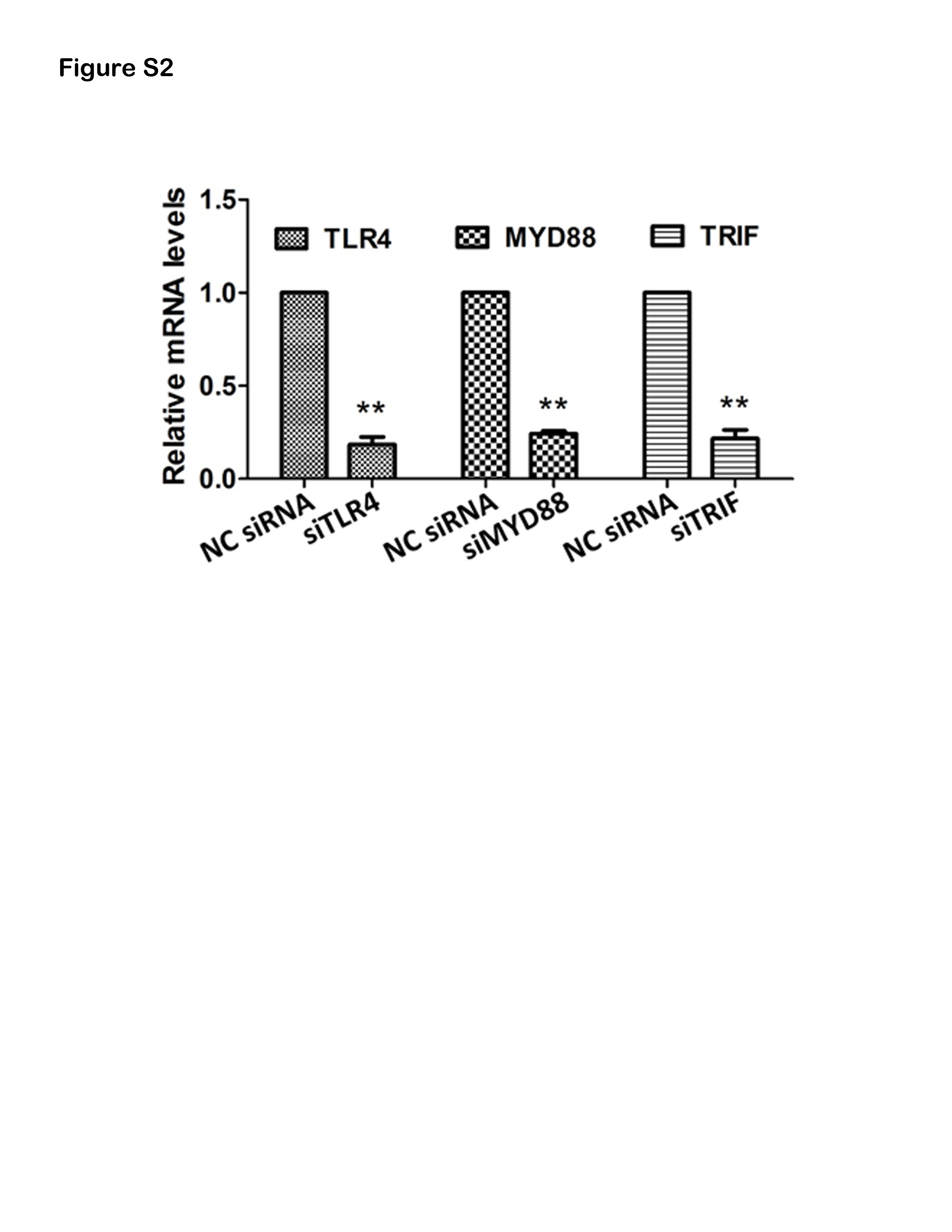

Supplement: Supplementary file 3 — Supplementary Figure S2 [file 41419_2020_2440_MOESM3_ESM.png]

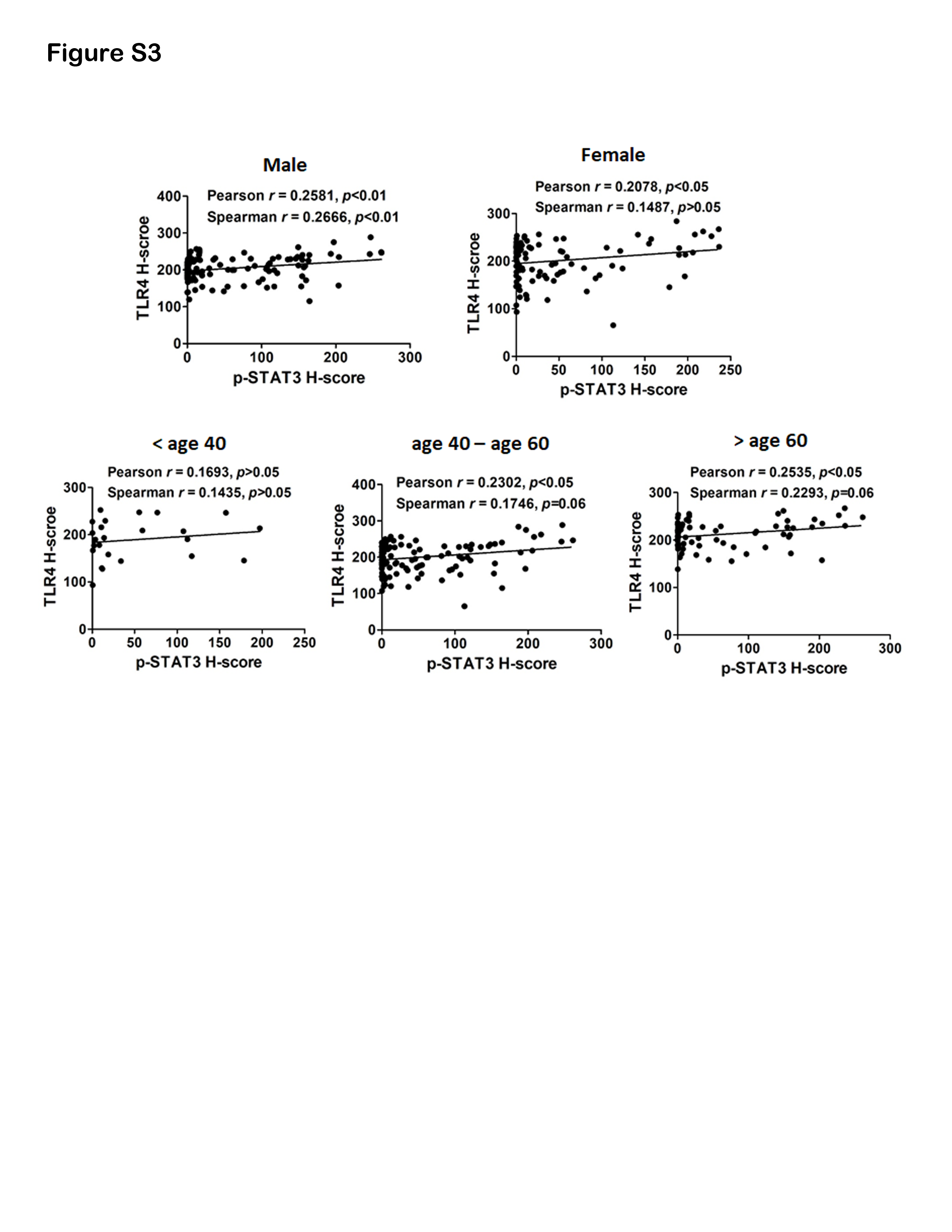

Supplement: Supplementary file 4 — Supplementary Figure S3 [file 41419_2020_2440_MOESM4_ESM.png]

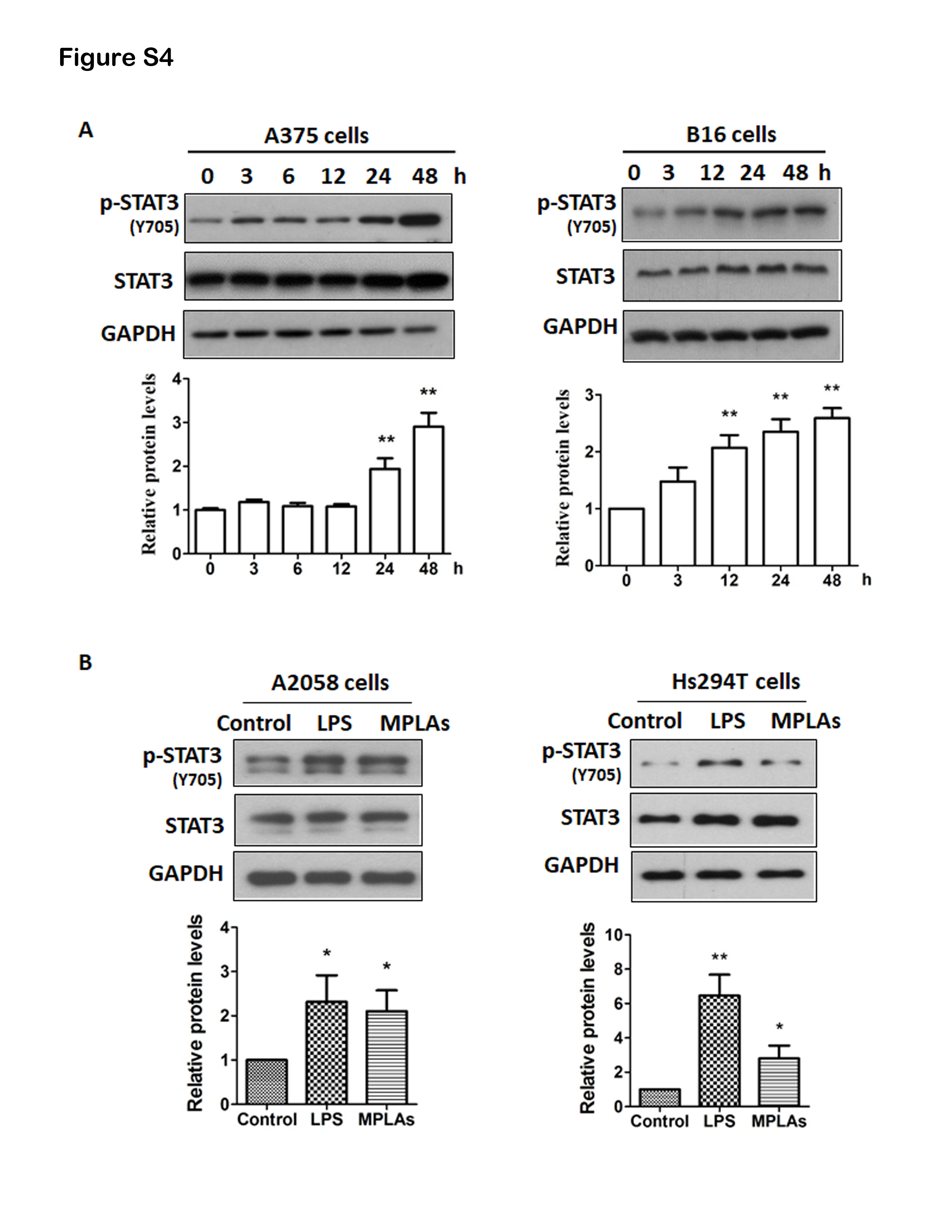

Supplement: Supplementary file 5 — Supplementary Figure S4 [file 41419_2020_2440_MOESM5_ESM.png]

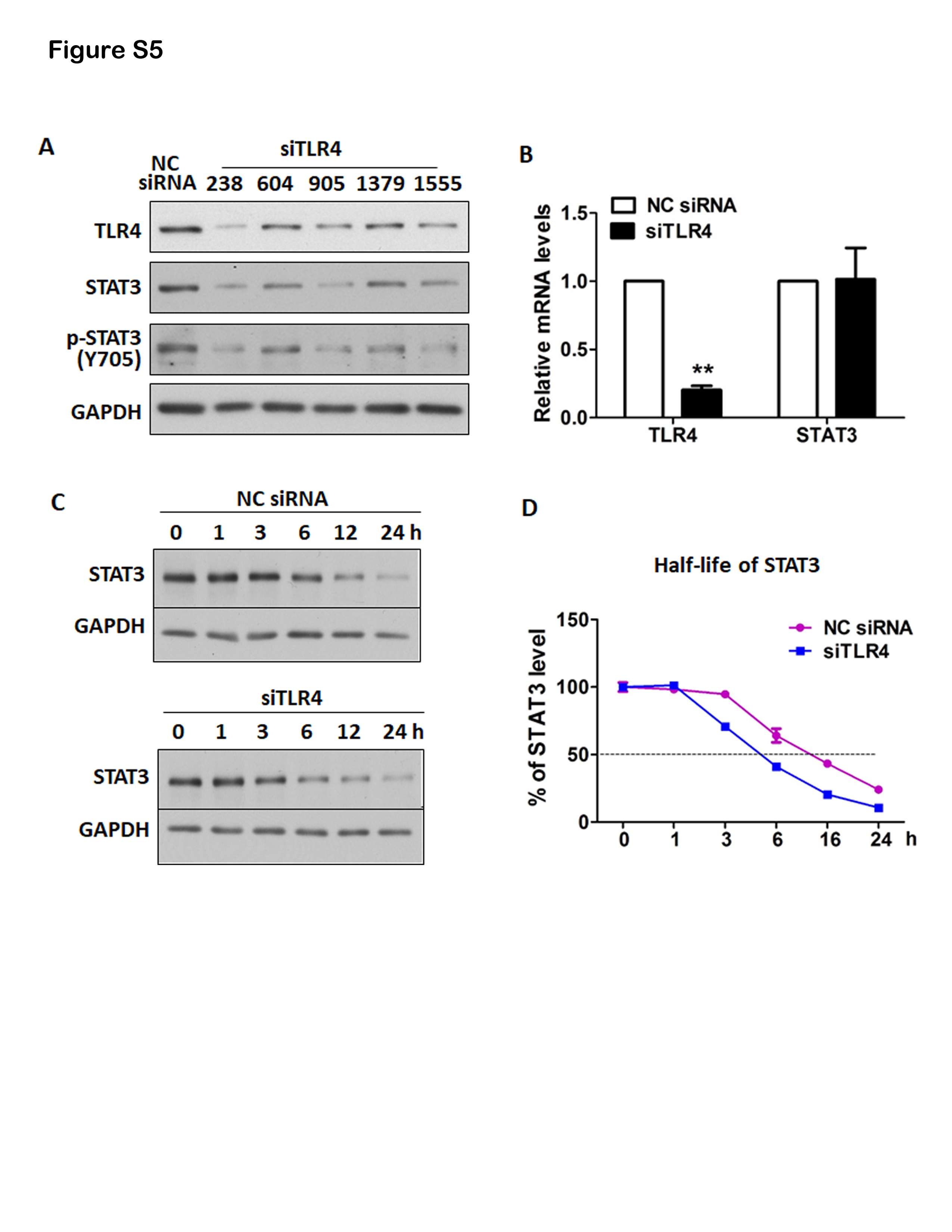

Supplement: Supplementary file 6 — Supplementary Figure S5 [file 41419_2020_2440_MOESM6_ESM.png]

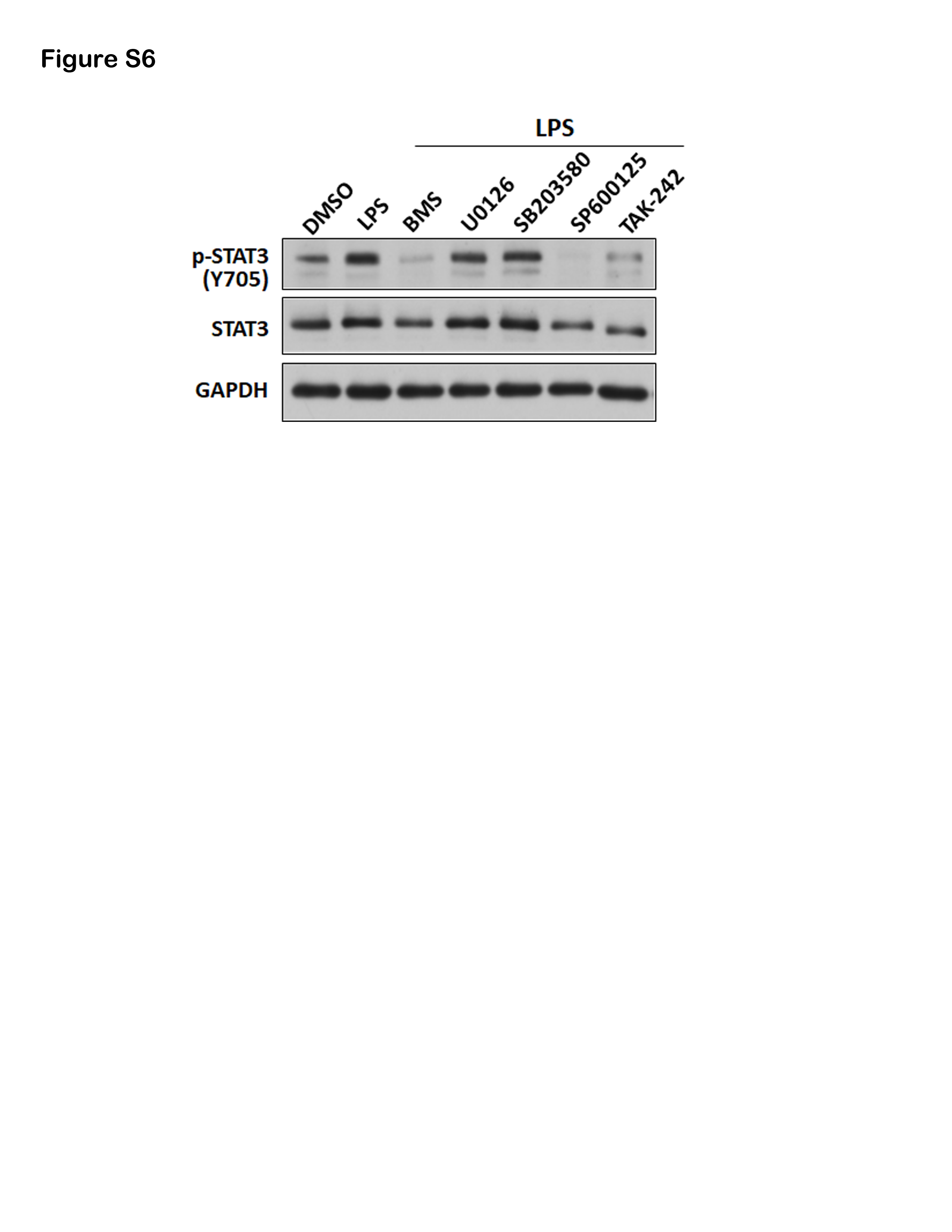

Supplement: Supplementary file 7 — Supplementary Figure S6 [file 41419_2020_2440_MOESM7_ESM.png]

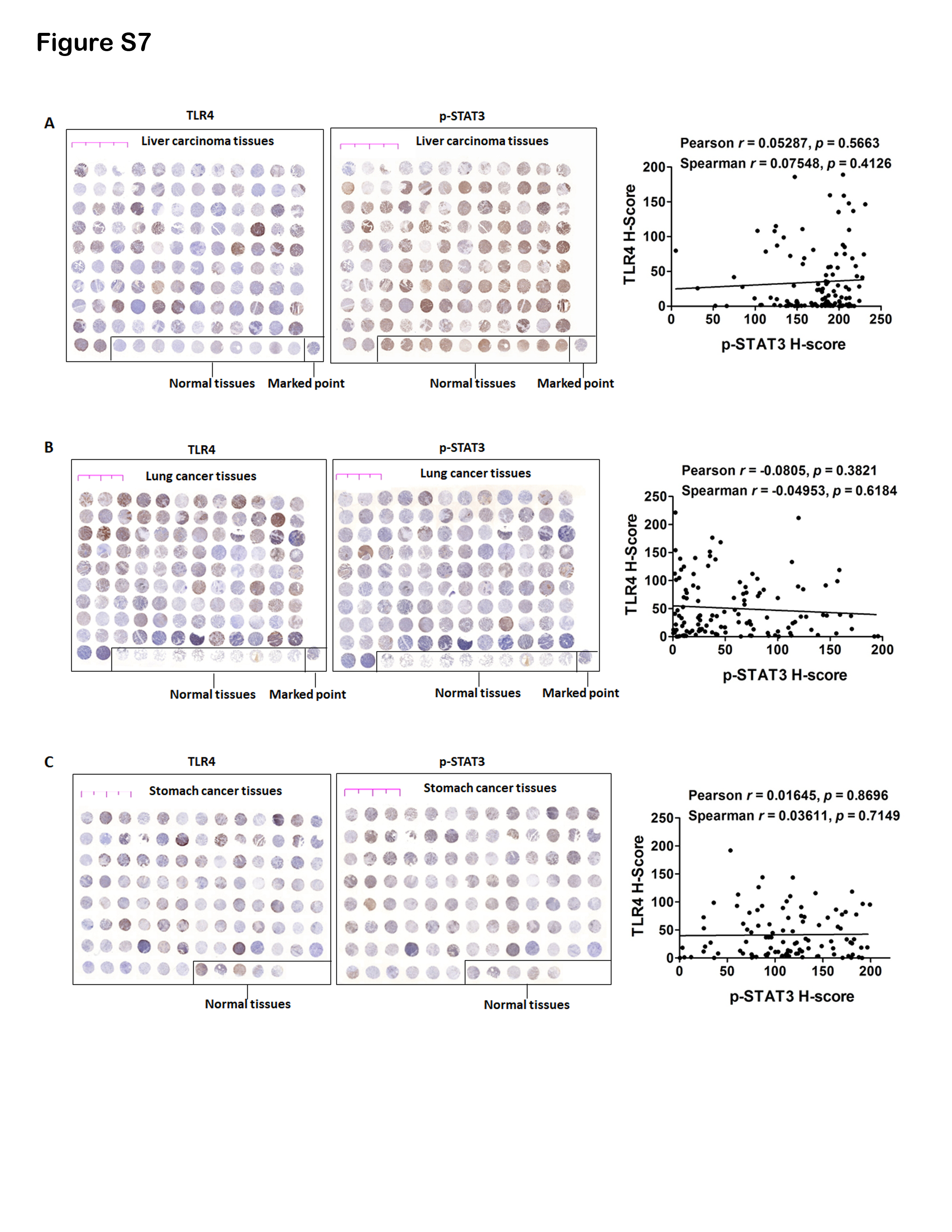

Supplement: Supplementary file 8 — Supplementary Figure S7 [file 41419_2020_2440_MOESM8_ESM.png]
