# Supplementary material for: Activation of STAT3 is a key event in TLR4 signaling-mediated melanoma progression
Source: Cell Death Dis. 2020 Apr 20;11(4):246. doi: 10.1038/s41419-020-2440-1 (PMC7171093; doi:10.1038/s41419-020-2440-1)
Supplement: Supplementary file 9 — Supplementary Tables [file 41419_2020_2440_MOESM9_ESM.docx]

**Table S1: Sequences of RT-qPCR primers.**

| **Gene** | **Primer Sequence** | |
| --- | --- | --- |
| **GAPDH** | Forward | GGTGTGAACCATGAGAAGTATGA |
|  | Reverse | GAGTCCTTCCACGATACCAAAG |
| **TLR4** | Forward | CCTAAGCCACCTCTCTACCTTA |
|  | Reverse | AGCCACCAGCTTCTGTAAAC |
| **MYD88** | Forward | GGTCCACCTCTCATCTGCAT |
|  | Reverse | GAAATGAAAAGCAGCCCAAG |
| **TRIF** | Forward | CCTCCTCCTTCATCTACTCCTT |
|  | Reverse | GCGTGGAGGATCACAAAGTTA |
| **MCL-1** | Forward | AAGCCAATGGGCAGGTCT |
|  | Reverse | TGTCCAGTTTCCGAAGCAT |
| **BCL-XL** | Forward | ATGAACTCTTCCGGGATGG |
|  | Reverse | TGGATCCAAGGCTCTAGGTG |
| **VEGF** | Forward | CACTGAGGAGTCCAACATCAC |
|  | Reverse | AGGAAGCTCATCTCTCCTATGT |
| **MMP2** | Forward | AAGTGGTCCGTGTGAAGTATG |
|  | Reverse | GGTATCAGTGCAGCTGTTGTA |
| **MMP9** | Forward | GAACTTTGACAGCGACAAGAAG |
|  | Reverse | CGGCACTGAGGAATGATCTAA |
| **STAT3** | Forward | GAGAAGGACATCAGCGGTAAG |
|  | Reverse | CAGTGGAGACACCAGGATATTG |

**Table S2**: **Sequences of TLR4 siRNAs.**

| **Name** | **Primer Sequence** | |
| --- | --- | --- |
| **238** | Sense (5’-3’) | CUGUGCAAUUUGACCAUUGTT |
|  | Antisense (5’-3’) | CAAUGGUCAAAUUGCACAGGC |
| **604** | Sense (5’-3’) | CCACCUCUCUACCUUAAUATT |
|  | Antisense (5’-3’) | UAUUAAGGUAGAGAGGUGGTT |
| **905** | Sense (5’-3’) | ACCUCUCUCAGUGUCAACUTT |
|  | Antisense (5’-3’) | AGUUGACACUGAGAGAGGUCC |
| **1379** | Sense (5’-3’) | UUCUUGCUGGCUGCAUAAATT |
|  | Antisense (5’-3’) | UUUAUGCAGCCAGCAAGAAGC |
| **1555** | Sense (5’-3’) | GGGCUUAGAACAACUAGAATT |
|  | Antisense (5’-3’) | UUCUAGUUGUUCUAAGCCCTT |

**Table S3: H-scores for evaluating TLR4 protein levels in melanoma samples from the tissue microarray.**

|  | **1** | **2** | **3** | **4** | **5** | **6** | **7** | **8** | **9** | **10** | **11** | **12** | **13** | **14** | **15** | **16** | **17** |
| --- | --- | --- | --- | --- | --- | --- | --- | --- | --- | --- | --- | --- | --- | --- | --- | --- | --- |
| **A** | 166.4 | 202.3 | 261.7 | 188.0 | 220.5 | 208.9 | 233.3 | 253.3 | 220.2 | 227.2 | 219.7 | 221.5 | 216.4 | 221.8 | 243.0 | 169.7 | X |
| **B** | 209.8 | 199.7 | 195.7 | 225.5 | 233.4 | 238.9 | 180.3 | 235.2 | 176.1 | 230.3 | 242.6 | 190.5 | 215.9 | 158.6 | 178.4 | 127.7 | X |
| **C** | 185.1 | 256.1 | 181.4 | 255.8 | 240.7 | 200.5 | 247.1 | 203.4 | 201.6 | 205.3 | 118.5 | 240.5 | 138.7 | 175.4 | 234.4 | 250.0 | X |
| **D** | 225.3 | 155.7 | 226.2 | 212.1 | 227.6 | 226.6 | 186.2 | 169.3 | 229.1 | 192.2 | 181.5 | 198.5 | 203.7 | 238.7 | 206.8 | 93.45 | X |
| **E** | 144.0 | 170.5 | 235.1 | 207.9 | 256.5 | 152.0 | 203.6 | 174.9 | 255.6 | 219.8 | 199.4 | 221.0 | 212.9 | 240.4 | 204.1 | 228.3 | X |
| **F** | 227.2 | 194.0 | 171.5 | 185.0 | 188.1 | 231.8 | 213.3 | 224.3 | 215.0 | 236.6 | 283.9 | 191.2 | 182.9 | 229.1 | 230.2 | 234.7 | X |
| **G** | 228.8 | 141.9 | 196.5 | 229.3 | 184.6 | 218.0 | 231.6 | 236.6 | 262.4 | 249.2 | 220.0 | 205.9 | 154.7 | 196.5 | 198.5 | 163.9 | X |
| **H** | 213.6 | 163.9 | 216.9 | 185.4 | 168.0 | 65.29 | 275.2 | 155.1 | 288.4 | 247.3 | 267.1 | 248.0 | 168.5 | 246.7 | 252.3 | 115.2 | X |
| **I** | 171.8 | 119.8 | 213.0 | 171.4 | 207.3 | 245.6 | 246.3 | 206.8 | 251.0 | 239.9 | 255.2 | 211.4 | 156.4 | 177.0 | 228.9 | 192.3 | X |
| **J** | 177.7 | 199.3 | 154.7 | 136.5 | 171.7 | 227.6 | 129.4 | 227.4 | 189.3 | 229.9 | 192.3 | 224.7 | 222.3 | 221.1 | 230.2 | 190.3 | X |
| **K** | 139.4 | 146.8 | 246.4 | 230.2 | 221.5 | 212.9 | 211.0 | 213.1 | 246.9 | 218.2 | 182.3 | 145.5 | 230.4 | 209.1 | 222.9 | 202.9 | X |
| **L** | 166.8 | 229.7 | 107.4 | 157.6 | 227.5 | 203.2 | 228.1 | 234.8 | 243.5 | 213.6 | 157.7 | 163.6 | 225.8 | 252.4 | 201.2 | 210.8 | X |
| **M** | 124.4 | 147.7 | 120.6 | 138.8 | 180.8 | 149.6 | 195.4 | 184.7 | 193.3 | 199.0 | 190.0 | 176.9 | 178.1 | 154.3 | 145.1 | 158.0 | 96.87 |

X, no sample.

**Table S4: H-scores for evaluating p-STAT3 (Y705) protein levels in melanoma samples from the tissue microarray.**

|  | **1** | **2** | **3** | **4** | **5** | **6** | **7** | **8** | **9** | **10** | **11** | **12** | **13** | **14** | **15** | **16** | **17** |
| --- | --- | --- | --- | --- | --- | --- | --- | --- | --- | --- | --- | --- | --- | --- | --- | --- | --- |
| **A** | 96.15 | 106.2 | 149.4 | 30.54 | 1.699 | 59.14 | 6.750 | 0.900 | 0.135 | 17.62 | 53.91 | 0.331 | 1.303 | 4.569 | 245.7 | 33.23 | X |
| **B** | 0.352 | 62.97 | 19.90 | 0.622 | 0.147 | 2.939 | 0.127 | 5.161 | 51.51 | 0.056 | 12.66 | 112.0 | 10.80 | 43.86 | 54.98 | 12.07 | X |
| **C** | 79.06 | 26.49 | 7.514 | 208.3 | 155.0 | 54.87 | 0.293 | 12.39 | 0.108 | 0.121 | 36.39 | 164.3 | 0.288 | 0.316 | 26.42 | 4.663 | X |
| **D** | 163.1 | 76.23 | 1.005 | 149.5 | 35.28 | 17.04 | 0.513 | 0.151 | 138.9 | 0.108 | 0.509 | 0.223 | 0.302 | 5.475 | 157.0 | 0.574 | X |
| **E** | 33.79 | 97.16 | 148.4 | 0.129 | 12.24 | 107.4 | 81.53 | 101.5 | 141.9 | 0.210 | 60.93 | 4.449 | 0.295 | 15.81 | 29.51 | 105.5 | X |
| **F** | 155.0 | 64.34 | 10.70 | 0.123 | 1.144 | 37.58 | 44.17 | 5.560 | 109.6 | 0.323 | 186.9 | 121.1 | 154.7 | 0.998 | 85.64 | 122.8 | X |
| **G** | 60.86 | 49.29 | 1.526 | 15.37 | 123.9 | 111.0 | 2.844 | 154.8 | 217.8 | 9.706 | 1.389 | 11.14 | 117.1 | 110.1 | 0.118 | 92.53 | X |
| **H** | 1.750 | 35.29 | 0.196 | 6.826 | 196.4 | 113.2 | 197.2 | 153.5 | 247.0 | 55.39 | 236.0 | 261.2 | 26.37 | 261.4 | 9.746 | 164.6 | X |
| **I** | 159.9 | 2.645 | 0.090 | 5.756 | 107.3 | 16.11 | 46.61 | 1.039 | 16.58 | 0.147 | 16.24 | 0.567 | 0.496 | 0.311 | 0.068 | 41.44 | X |
| **J** | 27.83 | 117.2 | 54.17 | 82.31 | 48.11 | 0.190 | 11.27 | 189.9 | 0.157 | 0.549 | 8.556 | 0.886 | 8.636 | 51.81 | 236.4 | 1.821 | X |
| **K** | 0.730 | 0.056 | 157.3 | 115.2 | 121.3 | 2.553 | 90.84 | 189.8 | 76.57 | 205.9 | 18.54 | 178.6 | 146.2 | 1.029 | 3.542 | 0.319 | X |
| **L** | 0.726 | 6.150 | 0.243 | 203.8 | 11.19 | 0.198 | 135.4 | 204.2 | 193.2 | 197.2 | **-** | 3.173 | 5.912 | 227.5 | 0.287 | 158.5 | X |
| **M** | 4.364 | 3.194 | 12.46 | 4.198 | 3.859 | **-** | 45.81 | 19.97 | 13.95 | 2.162 | 3.644 | 2.172 | 8.395 | 20.12 | 10.64 | 18.89 | 71.77 |

X, no sample. **-,** no positive signal.
